# Procedure time and 90-day outcomes after endovascular thrombectomy for large-core stroke

**DOI:** 10.1093/esj/aakag062

**Published:** 2026-06-26

**Authors:** Chengsong Yue, Shihai Yang, Rongzong Li, Linyu Li, Jie Yang, Xiaolei Shi, Wenjie Zi, Yi Lin

**Affiliations:** Department of Neurology and Institute of Neurology of The First Affiliated Hospital, Institute of Neuroscience, Fujian Key Laboratory of Molecular Neurology, Fujian Medical University, Fuzhou, Fujian 350005, China; Department of Neurology, National Regional Medical Center, Binhai Campus of the First Affiliated Hospital, Fujian Medical University, Fuzhou, Fujian 350212, China; Fujian Key Laboratory of Molecular Neurology, Institute of Neuroscience, Fujian Medical University, Fuzhou 350004, China; Department of Neurology, Xinqiao Hospital and The Second Affiliated Hospital, Army Medical University (Third Military Medical University), Chongqing, China; Department of Neurology, Xinqiao Hospital and The Second Affiliated Hospital, Army Medical University (Third Military Medical University), Chongqing, China; Department of Neurology, No. 924 Hospital of Joint Logistic Support Force of Chinese PLA, Guangxi 541002, China; Department of Neurology, Xinqiao Hospital and The Second Affiliated Hospital, Army Medical University (Third Military Medical University), Chongqing, China; Department of Neurology, Xinqiao Hospital and The Second Affiliated Hospital, Army Medical University (Third Military Medical University), Chongqing, China; Department of Neurology, Xinqiao Hospital and The Second Affiliated Hospital, Army Medical University (Third Military Medical University), Chongqing, China; Department of Neurology, Xinqiao Hospital and The Second Affiliated Hospital, Army Medical University (Third Military Medical University), Chongqing, China; Department of Neurology and Institute of Neurology of The First Affiliated Hospital, Institute of Neuroscience, Fujian Key Laboratory of Molecular Neurology, Fujian Medical University, Fuzhou, Fujian 350005, China; Department of Neurology, National Regional Medical Center, Binhai Campus of the First Affiliated Hospital, Fujian Medical University, Fuzhou, Fujian 350212, China; Fujian Key Laboratory of Molecular Neurology, Institute of Neuroscience, Fujian Medical University, Fuzhou 350004, China

**Keywords:** acute ischaemic stroke, dose–response relationship, EVT, large-core infarction, LVO, procedure time

## Abstract

**Introduction:**

Procedure time during endovascular thrombectomy (EVT) has been associated with outcomes in general thrombectomy cohorts, but whether this relationship reflects a threshold or continuous risk accumulation in large-core stroke remains unclear.

**Patients and methods:**

We performed a retrospective observational analysis of 490 patients with anterior-circulation large-vessel occlusion (LVO) and large-core infarction, defined by an Alberta Stroke Program Early Computed Tomography Score of 5 or less, from a prospective multicentre registry across 38 centres. Procedure time was analysed categorically and continuously. The primary outcome was the 90-day mRS score of 0–3. Secondary outcomes were 90-day mortality and sICH. Restricted cubic splines tested nonlinearity.

**Results:**

Each 10-min increase in procedure time was associated with lower odds of an modified Rankin Scale (mRS) score of 0–3 (odds ratio, 0.91; 95% CI, 0.86–0.95) and higher mortality (odds ratio, 1.07; 95% CI, 1.02–1.11). On the absolute scale, the marginally standardised probability of mRS 0–3 decreased from 44.5% at 60 min to 27.5% at 150 min (absolute risk difference − 17.1 percentage points; 95% CI, − 27.7 to −6.4). Spline analyses showed no evidence of nonlinearity for functional outcome or mortality. No consistent association with sICH was observed across analyses.

**Discussion:**

Longer procedure time was associated with progressively worse functional outcome and higher mortality in large-core EVT, without evidence of a clear nonlinear inflection point.

**Conclusion:**

These findings support procedure time as a continuous intraprocedural risk indicator that may inform ongoing procedural reassessment.

**Clinical trial registration:**

MAGIC registry, ChiCTR.org.cn Identifier: ChiCTR2100051664.

## Graphical Abstract

**Figure:**
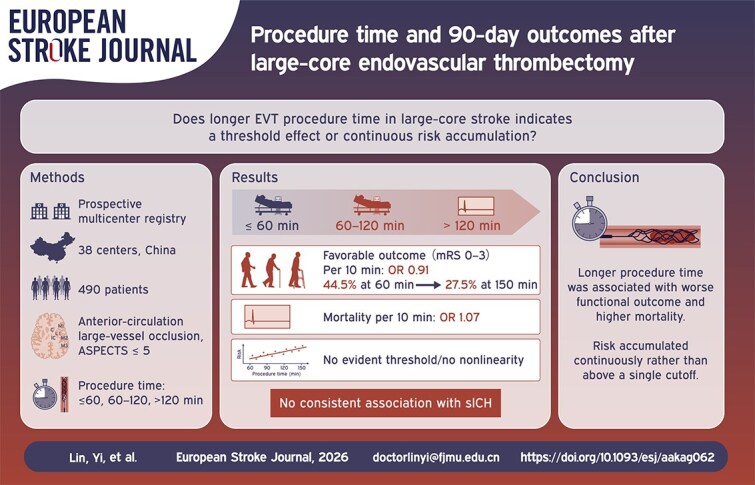

## Introduction

Endovascular thrombectomy (EVT) is the standard of care for anterior-circulation large-vessel occlusion (LVO) stroke.^1^ Treatment delay—from symptom onset or last-known-well to arterial puncture and reperfusion—is consistently associated with worse functional outcomes and higher mortality.^2–5^ Although most time-related research has focused on preprocedural intervals, procedure time (PT), defined as the interval from groin puncture to reperfusion or procedure termination,^6^ represents a distinct intraprocedural time interval that accumulates after the treatment decision has already been made. In general EVT cohorts, longer PT has been associated with worse 90-day outcomes even after accounting for preprocedural delays such as onset-to-puncture time,^5^^,^^7^ suggesting that the duration of the procedure itself may carry prognostic information beyond that captured by workflow metrics alone.

Recent randomised trials, including RESCUE-Japan LIMIT, ANGEL-ASPECT, SELECT2 and TENSION, demonstrated functional benefit of EVT in selected patients with large-core stroke, thereby extending thrombectomy to a population with substantial baseline tissue injury and limited physiologic reserve.^8–11^ As EVT for large-core stroke enters routine clinical practice, attention has increasingly turned to procedural factors beyond final reperfusion that may influence outcomes in this highly vulnerable population.^12^^,^^13^ In mixed or standard-core EVT populations, studies have reported both potential time thresholds, such as a “golden hour,”^14^ and progressively worse outcomes with longer PT that are more consistent with continuous risk accumulation.^5^^,^^15^ However, these findings cannot be directly extrapolated to large-core patients, in whom the dose–response shape of PT remains insufficiently characterised. A recent secondary analysis of SELECT2 linked longer PT to a worse 90-day modified Rankin Scale (mRS) distribution in large-core patients and quantified incremental risk per 10-min increase, but did not formally test for nonlinearity or characterise the continuous dose–response curve; moreover, the absolute magnitude of PT-associated risk in real-world large-core practice remains uncertain.^16^

We therefore used data from the MAGIC registry, a prospective multicentre cohort of patients with anterior-circulation LVO and large-core infarction defined by noncontrast CT Alberta Stroke Program Early CT Score (ASPECTS) ≤ 5 across 38 Chinese stroke centres, to evaluate the association between PT and 90-day outcome after attempted EVT. Specifically, we sought to characterise the dose–response shape of this association and to quantify the clinical magnitude of PT-associated risk across the observed PT range.

## Patients and methods

### Study design, setting and data source

This was a retrospective observational cohort analysis of prospectively collected data from the MAGIC registry (ChiCTR2100051664), a nationwide prospective multicentre registry conducted at 38 stroke centres in China from November 2021 through February 2023. Although the registry was prospectively established, the present analysis was conducted retrospectively to evaluate the association between PT and 90-day outcomes among patients undergoing attempted EVT. This study is reported in accordance with the STROBE statement.^17^

### Participants

**Figure 1:**
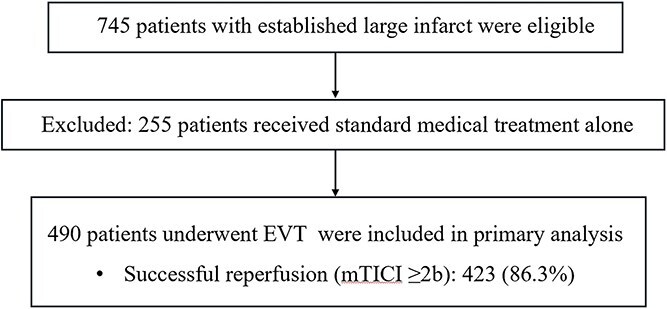
Study flow diagram. The flow diagram shows patient selection from the source cohort of patients with established large infarction. Of 745 eligible patients, 255 who received standard medical treatment alone were excluded, and 490 who underwent EVT were included in the primary analysis. Successful reperfusion (mTICI grade ≥ 2b) was achieved in 423 patients (86.3%). Abbreviation: mTICI, modified thrombolysis in cerebral infarction.

The MAGIC registry enrolled patients with acute ischaemic stroke due to anterior-circulation LVO (intracranial internal carotid artery or M1/M2 middle cerebral artery) with established large infarction on noncontrast CT (ASPECTS ≤ 5), presenting within 24 h of symptom onset or last known-well time.^18^ Registry exclusions included prestroke disability (mRS > 2), life-threatening comorbidities unrelated to stroke and inability to obtain 90-day outcome assessment.^18^ For the present analysis, we included all patients who underwent attempted EVT, yielding an analytic cohort of 490 patients (Figure 1). This analytic cohort corresponds to the EVT-treated subgroup of the previously reported MAGIC large-core cohort, in which 490 patients underwent attempted EVT plus standard medical therapy and 255 patients received standard medical therapy alone. Patients receiving standard medical therapy alone in the parent cohort were not eligible for the present PT analysis because PT, defined from groin puncture to reperfusion or procedure termination, was not applicable to them. Successful reperfusion (mTICI 2b–3) was not used as an inclusion criterion in the primary analysis to avoid conditioning on a post-exposure procedural outcome.^19^

All EVT procedures were performed at participating stroke centres by experienced neurointerventionists following institutional standard-of-care protocols.

### Data collection and quality control

Baseline variables included demographics, vascular risk factors, prestroke functional status and stroke severity measured by the NIHSS.^20^ Imaging variables included ASPECTS,^21^ occlusion site and collateral status graded by the modified American Society of Interventional and Therapeutic Neuroradiology/Society of Interventional Radiology collateral grading system (ASITN/SIR) scale.^22^ Stroke aetiology was classified using the TOAST system.^23^ Additional workflow and procedural variables, including onset-to-imaging time, onset-to-puncture time, intravenous thrombolysis, anaesthesia modality, thrombectomy technique and number of passes, were collected for descriptive analyses and imputation models.

Imaging parameters, including baseline infarct extent (ASPECTS), occlusion site, angiographic reperfusion grade and ICH/haemorrhagic transformation on follow-up imaging, were independently adjudicated by trained readers according to prespecified criteria, with readers blinded to 90-day clinical outcomes. Reperfusion was graded using the modified thrombolysis in cerebral infarction (mTICI) scale.^24^

### Exposure: procedure time

Procedure time was the primary exposure of interest. When successful reperfusion was achieved, PT was defined as the interval from groin puncture to the time of first achieving mTICI ≥ 2b; when reperfusion was not achieved, PT was defined as groin puncture to procedure termination or final angiographic assessment.^25^^,^^26^ For patients without successful reperfusion, PT therefore represents total procedural exposure duration rather than time to first substantial reperfusion. The primary analysis included all patients regardless of reperfusion status, and a prespecified sensitivity analysis restricted to patients achieving successful reperfusion (mTICI ≥ 2b) was also performed (Methods S6).

Procedure time was modelled in 2 ways: (1) as a 3-category exposure (≤60, > 60–≤120 and > 120 min; reference, ≤ 60 min), consistent with categorisations used in prior EVT analyses,^27^^,^^28^ and (2) as a continuous exposure per 10-min increase.

### Outcomes

Ninety-day outcomes were obtained using standardised follow-up procedures (in-person assessment or structured telephone interview). The primary outcome was 90-day favourable functional outcome, defined as an mRS score of 0–3, consistent with recent large-core infarction trials and prior analyses in this population.^8^^,^^18^ Secondary outcomes included 90-day all-cause mortality, sICH within 48 h according to the Heidelberg Bleeding Classification,^29^ and additional dichotomised mRS outcomes (mRS 0–2 and mRS 0–4). The full ordinal distribution of 90-day mRS (0–6) was additionally evaluated using shift analysis.^30^^,^^31^

### Statistical analysis

All analyses were performed in R version 4.3.1. Continuous variables are summarised as mean (SD) or median (IQR), and categorical variables as counts and percentages. All statistical tests were 2-sided, and *P* < .05 was considered statistically significant.

### Confounding control and centre effects

A prespecified directed acyclic graph (DAG; Methods S1 and Figure S1) guided covariate selection.^32^ The DAG identified age, baseline NIHSS, ASPECTS, collateral grade (modified ASITN/SIR), occlusion site and TOAST subtype as common causes of both PT and outcome and therefore as the core confounder set for primary adjustment. Onset-to-puncture time was not included in the primary adjustment set because its effect on outcome was considered to be predominantly mediated through baseline infarct severity at the time of puncture, reflected by NIHSS, ASPECTS and collateral status; it was retained as an auxiliary variable in the imputation model. Intraprocedural variables such as number of passes, rescue techniques and anaesthesia modality were considered potential mediators or descendants of the exposure and were therefore excluded from primary models to avoid overadjustment.^33^

Primary models adjusted for age, baseline NIHSS, ASPECTS, collateral grade, occlusion site and TOAST subtype (model 1). Expanded models additionally adjusted for sex and admission glucose (model 2), both established prognostic factors in ischaemic stroke and EVT cohorts,^34–36^ to improve precision and assess robustness to expanded covariate control.

Between-centre clustering was addressed using a random intercept for centre in fully adjusted unweighted models (Methods S3).^37^^,^^38^ In propensity score-weighted analyses, centre was modelled as a fixed effect because random-intercept estimation can be unstable when combined with inverse-probability weights, particularly after weight truncation and in the presence of small within-centre sample sizes.^39^^,^^40^

Binary outcomes were analysed using logistic regression and reported as odds ratios (ORs) with 95% CIs. Models were fitted as an unadjusted model, model 1, model 2 and model 3 (model 2 plus a random intercept for centre using mixed-effects logistic regression) (Methods S3). The ordinal 90-day mRS outcome (0–6) was analysed using cumulative logit proportional-odds models; for model 3, a mixed-effects proportional-odds model with a random centre intercept was used with the same covariate strategy and centre handling (Methods S3).

### Missing data and multiple imputation

Missing values in PT, admission glucose, systolic blood pressure, diastolic blood pressure and onset-to-puncture time were imputed under a missing-at-random assumption^41^ using multiple imputation by chained equations with predictive mean matching (20 imputations; 20 iterations).^42^ Systolic and diastolic blood pressure were included in the imputation model as auxiliary variables to improve imputation quality but were not included in outcome models. Outcome variables were included as auxiliary variables in the imputation model but were not themselves imputed.^41^ All analyses were performed within each imputed dataset and pooled using Rubin’s rules.^43^ Additional details are provided in Methods S2, Table S1 and Figures S2–S4. Descriptive summaries by PT category were based on observed PT values (*n* = 484), whereas inferential analyses used multiple imputation to retain the full cohort (*n* = 490).

### Dose–response shape and nonlinearity assessment

For the primary outcome and 90-day mortality, the dose–response shape of PT was characterised by modelling PT per 10 min using a 4-knot restricted cubic spline in fully adjusted mixed-effects models with a random intercept for centre (Methods S5).^44^^,^^45^ Adjusted ORs were referenced to PT = 60 min, corresponding to the upper boundary of the reference category in the categorical analysis, to facilitate comparison across modelling approaches. Nonlinearity was evaluated using a joint Wald test of the nonlinear spline terms. To improve clinical interpretability, we additionally reported marginally standardised adjusted probabilities and absolute risk differences at prespecified PT values (60, 90, 120 and 150 min) (Table 3). Adjusted predicted probability curves across PT are provided in Figure S5.

### Propensity score weighting and doubly robust estimation

For the 3-category PT exposure, generalised propensity scores were estimated using multinomial logistic regression with model 2 covariates.^46^ Stabilised average treatment effect weights were truncated at the 1st and 99th percentiles.^47^ Weighted outcome models additionally adjusted for the same covariates to obtain doubly robust estimates,^48^ with centre included as a fixed effect. Covariate balance was assessed using standardised mean differences and Love plots (Methods S4; Tables S2 and S3; Figure S6).^49^

### Sensitivity analyses

Sensitivity analyses included: (1) complete-case analyses in the overall EVT cohort, restricted to patients with non-missing values for the exposure and all covariates included in the corresponding adjusted models (*n* = 472; Tables S4 and S5); (2) complete-case analyses restricted to patients achieving successful reperfusion (mTICI ≥ 2b; *n* = 408; Tables S6 and S7), to assess whether associations persisted after angiographic success and (3) assessment of the proportional-odds assumption for ordinal mRS models. For the ordinal mRS shift outcome in the overall EVT cohort, the proportional-odds assumption was evaluated using the Brant test^50^ in model 2 without centre terms, and a cumulative link mixed model with a random centre intercept was fitted to account for centre-level clustering (Methods S6).

### Exploratory analyses

We performed 2 additional exploratory analyses: subdivision of the original ≤ 60-min PT reference category into PT < 30 min vs PT 30–60 min, and a PT-by-collateral interaction analysis in the overall EVT cohort using poor (ASITN/SIR 0–1) vs better (ASITN/SIR 2–4) collaterals. Detailed model specifications are provided in Methods S7. These analyses were exploratory and were not used to redefine the primary PT categories.

### Ethics

The registry was approved by the ethics committee of Xinqiao Hospital, Army Medical University (approval No. 2021-yandi 134-01) and the institutional review boards of all participating sites. Written informed consent was obtained from patients or their legally authorised representatives.

## Results

### Cohort and procedure time distribution

In the nationwide prospective multicentre MAGIC registry (38 stroke centres; November 2021 to February 2023), 490 patients with anterior-circulation LVO and large-core infarction (ASPECTS ≤ 5) underwent attempted EVT. Procedure time was observed in 484 patients and imputed for the remaining 6. Median PT was 71 min (IQR, 51–115). Patients were categorised into PT ≤ 60 min (*n* = 185), PT > 60–≤120 min (*n* = 197) and PT > 120 min (*n* = 102) (Table 1). Several baseline and procedural characteristics differed across PT strata, including stroke mechanism and occlusion site distribution (Table 1).

**Table 1:** Baseline characteristics of all EVT patients, stratified by PT groups.

	Fast group (*n* = 185)	Intermediate group (*n* = 197)	Extended group (*n* = 102)
**Age, y, median (IQR)**	69.0 (59.0, 78.0)	69.0 (60.0, 77.0)	70.5 (57.0, 78.0)
**Sex, male, *n* (%)**	109 (58.9)	110 (55.8)	61 (59.8)
**Glucose, mmol/L, median (IQR)**	6.9 (5.8, 8.6)	7.6 (6.2, 9.1)	7.0 (5.9, 9.1)
**BP, mmHg, median (IQR)**			
** Systolic**	142.0 (128.0, 159.0)	150.0 (133.0, 170.0)	143.0 (123.0, 160.0)
** Diastolic**	85.0 (75.0, 96.0)	87.0 (76.0, 97.0)	84.0 (75.0, 92.0)
**Medical history, *n* (%)**			
** Hypertension**	114 (61.6)	118 (59.9)	62 (60.8)
** Hyperlipidaemia**	46 (24.9)	39 (19.8)	20 (19.6)
** Diabetes**	29 (15.7)	31 (15.7)	11 (10.8)
** Smoking**	63 (34.1)	57 (28.9)	31 (30.4)
** Atrial fibrillation**	94 (50.8)	85 (43.1)	39 (38.2)
**ASPECTS, median (IQR)**	4 (2, 5)	4 (2, 5)	4 (3, 5)
**NIHSS score, median (IQR)**	18 (14, 20)	17 (14, 20)	16 (13, 21)
**ASITN/SIR, *n* (%)**			
** 0–1**	94 (50.8)	98 (49.7)	44 (43.1)
** 2**	61 (33.0)	66 (33.5)	39 (38.2)
** 3–4**	30 (16.2)	33 (16.8)	19 (18.6)
**TOAST, *n* (%)**			
** LAA**	38 (20.5)	65 (33.0)	40 (39.2)
** CE**	125 (67.6)	109 (55.3)	40 (39.2)
** Others/unknown**	22 (11.9)	23 (11.7)	22 (21.6)
**Occlusion site, *n* (%)**			
** ICA**	68 (36.8)	87 (44.2)	47 (46.1)
** M1 segment**	101 (54.6)	93 (47.2)	37 (36.3)
** M2 segment**	16 (8.6)	17 (8.6)	18 (17.6)
**First choice of EVT, *n* (%)**			
** Stent thrombectomy**	62 (33.5)	74 (37.6)	42 (41.2)
** Aspiration**	109 (58.9)	107 (54.3)	50 (49.0)
** Others**	14 (7.6)	16 (8.1)	10 (9.8)
**IVT, *n* (%)**	41 (22.2)	47 (23.9)	30 (29.4)
**OTI, min, median (IQR)**	286.0 (175.0, 399.0)	317.0 (183.0, 523.0)	234.0 (109.0, 410.0)
**OTP, min, median (IQR)**	362.0 (240.0, 500.0)	380.0 (256.0, 610.0)	351.5 (198.0, 530.0)
**Anaesthesia, general, *n* (%)**	40 (21.6)	20 (10.2)	24 (23.5)

Abbreviations: ASITN/SIR = American Society of Interventional and Therapeutic Neuroradiology/Society of Interventional Radiology collateral grading system; ASPECTS = Alberta Stroke Program Early CT Score; BP = blood pressure; CE = cardioembolism; IVT = intravenous thrombolysis; LAA, large-artery atherosclerosis; OTI = onset-to-imaging time; OTP = onset-to-puncture time; PT = procedure time; TOAST = Trial of Org 10172 in Acute Stroke Treatment classification.

Data are median (IQR) or number (percentage), as appropriate, and are based on the original analytic cohort before multiple imputation.

No.(%) were calculated among patients with observed PT (*n* = 484). Patients were stratified into 3 groups according to PT: Fast group (PT ≤ 60 min), Intermediate group (PT > 60–≤120 min) and Extended group (PT > 120 min).

### Procedure time and 90-day functional outcome

When modelled continuously, each 10-min increase in PT was associated with lower odds of achieving 90-day mRS 0–3 (model 3: OR 0.91; 95% CI, 0.86–0.95; *P* < .001) (Table 2). Restricted cubic spline analyses showed no evidence of nonlinearity for mRS 0–3 (*P* for nonlinearity = .737) (Figure 3), and adjusted predicted probabilities across PT are shown in Figure S5.

**Table 2:** Association of PT (categorical and continuous) with 90-day outcomes among patients undergoing EVT.

Outcome			No (%)	Crude OR	*P* value	Model 1		Model 2		Model 3	
						Adjusted OR	*P* value	Adjusted OR	*P* value	Adjusted OR	*P* value
**Primary outcome**											
**mRS 0–3**											
	Categorical PT	≤60 min	83/185 (44.9)	Reference	—	Reference	—	Reference	—	Reference	—
		>60– ≤ 120 min	63/197 (32.0)	0.59 (0.39, 0.89)	0.012	0.48 (0.29, 0.79)	.004	0.49 (0.30, 0.82)	.006	0.48 (0.28, 0.81)	.006
		>120 min	32/102 (31.4)	0.56 (0.34, 0.93)	0.026	0.34 (0.18, 0.65)	.001	0.33 (0.17, 0.63)	<.001	0.30 (0.15, 0.60)	<.001
		*P* for trend	*P* value < .001								
	Continuous PT	Per 10 min	—	0.95 (0.92, 0.99)	0.006	0.92 (0.87, 0.96)	<.001	0.91 (0.87, 0.96)	<.001	0.91 (0.86, 0.95)	<.001
**Secondary outcome**											
**mRS 0–2**											
	Categorical PT	≤60 min	49/185 (26.5)	Reference	—	Reference	—	Reference	—	Reference	—
		>60– ≤ 120 min	38/197 (19.3)	0.67 (0.42, 1.09)	0.104	0.67 (0.40, 1.14)	.138	0.70 (0.41, 1.20)	.196	0.70 (0.41, 1.21)	.203
		>120 min	16/102 (15.7)	0.52 (0.28, 0.97)	0.041	0.44 (0.22, 0.90)	.024	0.44 (0.21, 0.90)	.024	0.42 (0.20, 0.88)	.022
		*P* for trend	*P* value = .020								
	Continuous PT	Per 10 min	—	0.95 (0.91, 0.99)	0.026	0.94 (0.89, 0.99)	.017	0.94 (0.89, 0.99)	.015	0.94 (0.89, 0.99)	.013
**mRS 0–4**											
	Categorical PT	≤60 min	104/185 (56.2)	Reference	—	Reference	—	Reference	—	Reference	—
		>60− ≤ 120 min	92/197 (46.7)	0.69 (0.46, 1.04)	0.074	0.60 (0.37, 0.98)	.04	0.63 (0.38, 1.04)	.071	0.63 (0.38, 1.04)	.071
		>120 min	48/102 (47.1)	0.71 (0.44, 1.16)	0.171	0.47 (0.25, 0.87)	.016	0.44 (0.23, 0.84)	.012	0.44 (0.23, 0.84)	.012
		*P* for trend	*P* value = .009								
	Continuous PT	Per 10 min	—	0.97 (0.94, 1.00)	0.072	0.95 (0.91, 0.99)	.007	0.94 (0.90, 0.98)	.003	0.94 (0.90, 0.98)	.003
**mRS 0–6 (median [IQR])**											
	Categorical PT	≤60 min	4 (2,6)	Reference	—	Reference	—	Reference	—	Reference	—
		>60− ≤ 120 min	5 (3,6)	1.53 (1.06, 2.20)	0.022	1.59 (1.08, 2.33)	.019	1.53 (1.04, 2.26)	.031	1.54 (1.03, 2.31)	.035
		>120 min	5(3,6)	1.64 (1.06, 2.54)	0.026	2.17 (1.33, 3.53)	.002	2.25 (1.37, 3.68)	.001	2.36 (1.40, 3.98)	.001
		*P* for trend	*P* value < .001								
	Continuous PT	Per 10 min	—	1.04 (1.01, 1.07)	0.011	1.06 (1.03, 1.09)	<.001	1.07 (1.03, 1.10)	<.001	1.07 (1.04, 1.11)	<.001
**Mortality**											
	Categorical PT	≤60 min	68/185 (36.8)	Reference	—	Reference	—	Reference	—	Reference	—
		>60− ≤ 120 min	88/197 (44.7)	1.36 (0.91, 2.05)	0.136	1.55 (0.95, 2.50)	.077	1.44 (0.88, 2.38)	.149	1.49 (0.88, 2.51)	.136
		>120 min	48/102 (47.1)	1.48 (0.91, 2.41)	0.116	2.27 (1.24, 4.17)	.008	2.39 (1.27, 4.47)	.007	2.51 (1.29, 4.88)	.007
		*P* for trend	*P* value = .007								
	Continuous PT	Per 10 min	—	1.03 (1.00, 1.06)	0.062	1.06 (1.02, 1.10)	.005	1.06 (1.02, 1.11)	.002	1.07 (1.02, 1.11)	.002
**sICH**											
	Categorical PT	≤60 min	21/185 (11.4)	Reference	—	Reference	—	Reference	—	Reference	—
		>60– ≤ 120 min	28/197 (14.2)	1.30 (0.71, 2.37)	0.401	1.34 (0.72, 2.52)	.357	1.22 (0.64, 2.31)	.545	1.22 (0.64, 2.31)	.545
		>120 min	16/102 (15.7)	1.44 (0.72, 2.91)	0.304	1.50 (0.71, 3.17)	.293	1.53 (0.71, 3.27)	.274	1.53 (0.71, 3.27)	.274
		*P* for trend	*P* value = .274								
	Continuous PT	Per 10 min	—	1.02 (0.98, 1.06)	0.326	1.03 (0.98, 1.07)	.245	1.03 (0.98, 1.08)	.201	1.03 (0.98, 1.08)	.201

Abbreviations: ASPECTS = Alberta Stroke Program Early CT Score; ASITN/SIR = American Society of Interventional and Therapeutic Neuroradiology/Society of Interventional Radiology collateral grading system; OR = odds ratio; PT = procedure time; TOAST = Trial of Org 10172 in Acute Stroke Treatment classification.

Data are ORs with 95% CIs from models fitted within the multiply imputed analytic dataset (20 imputations; *n* = 490) and pooled using Rubin’s rules.

The “No./Total (%) or Median (IQR)” column was calculated among patients with observed PT (*n* = 484).

For dichotomous functional outcomes (mRS 0–2, 0–3 and 0–4) and safety outcomes (mortality and sICH), the event was defined as a favourable outcome for functional endpoints and the occurrence of the adverse event for safety endpoints; thus, for functional outcomes ORs < 1 indicate lower odds of achieving a favourable outcome with longer PT, whereas for mortality and sICH ORs > 1 indicate increased odds of the respective adverse outcome.

For the ordinal 90-day mRS (0–6) shift outcome, ORs are from cumulative logit proportional-odds models; ORs > 1 indicate a shift towards higher (worse) mRS categories with longer PT.

PT was analysed as either a categorical exposure (≤60, > 60–≤120 and > 120 min; reference ≤ 60 min) or a continuous exposure per 10-min increase (PT/10).

“Crude” denotes unadjusted models including PT only.

Model 1 was adjusted for age, baseline NIHSS score, ASPECTS, ASITN/SIR collateral grade, occlusion site and TOAST subtype.

Model 2 was additionally adjusted for sex and admission glucose.

Model 3 additionally included a random intercept for centre (mixed-effects logistic regression for binary outcomes and mixed-effects proportional-odds models for the ordinal mRS shift outcome).

*P* for trend was obtained from a Wald test by modelling PT category as an ordinal variable (1, 2, 3) using the same fixed- and random-effects structure as model 3.

**Figure 2:**
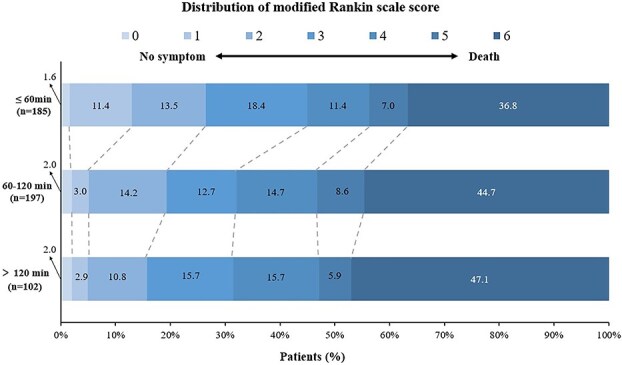
Distribution of 90-day mRS scores by PT category. Stacked bars show the distribution of 90-day mRS scores (0–6) across PT categories among patients with observed PT (≤60 min, *n* = 185; > 60–≤120 min, *n* = 197; > 120 min, *n* = 102). Six patients with missing PT were excluded from this descriptive figure; 90-day mRS was complete. The proportion of the primary outcome (mRS 0–3) was lower in the longer-PT groups (44.9%, 32.0% and 31.4%, respectively), while the proportion of death (mRS = 6) increased (36.8%, 44.7% and 47.1%). Percentages are calculated within each PT category and may not sum to 100% due to rounding. Abbreviation: PT  =  procedure time.

Categorical analyses corroborated this gradient. The proportion of patients achieving mRS 0–3 at 90 days was 44.9% (83/185) in the PT ≤ 60-min group, 32.0% (63/197) in the PT > 60–≤120-min group and 31.4% (32/102) in the PT > 120-min group (Table 2; Figure 2). In fully adjusted mixed-effects models including a random intercept for centre (model 3), both intermediate PT (OR 0.48; 95% CI, 0.28–0.81; *P* = .006) and extended PT (OR 0.30; 95% CI, 0.15–0.60; *P* < .001) were associated with lower odds of achieving mRS 0–3 compared with PT ≤ 60 min, with a monotonic trend across PT strata (*P* for trend < .001).

To translate this continuous association onto an absolute-risk scale, marginally standardised adjusted probabilities and absolute risk differences (ARDs) at selected PT values are summarised in Table 3. The adjusted probability of mRS 0–3 decreased from 44.5% at 60 min to 38.3% at 90 min, 32.2% at 120 min and 27.5% at 150 min (ARD at 150 vs 60 min: −17.1 percentage points; 95% CI, −27.7 to −6.4).

**Table 3:** Marginally standardised adjusted probabilities and absolute risk differences at key PTs for 90-day mRS 0–3 and mortality.

Procedure time (minutes)	mRS 0–3 at 90 days		Mortality at 90 days	
	Adjusted probability (95% CI)	ARD vs 60 min, percentage points (95% CI)	Adjusted probability (95% CI)	ARD vs 60 min, percentage points (95% CI)
**60**	44.5% (36.8–52.2)	Reference	38.8% (31.3–46.2)	Reference
**90**	38.3% (31.8–44.9)	−6.2 (−11.8, −0.5)	40.9% (34.3–47.5)	2.1 (−2.9, 7.2)
**120**	32.2% (24.6–39.8)	−12.3 (−22.2, −2.5)	42.5% (34.6–50.4)	3.8 (−5.7, 13.2)
**150**	27.5% (19.8–35.2)	−17.1 (−27.7, −6.4)	45.2% (36.4–54.0)	6.4 (−4.6, 17.5)

Abbreviations: ARD = absolute risk difference; ASITN/SIR = American Society of Interventional and Therapeutic Neuroradiology/Society of Interventional Radiology collateral grading system; ASPECTS = Alberta Stroke Program Early CT Score; PT = procedure time; TOAST = Trial of Org 10172 in Acute Stroke Treatment.

Values are marginally standardised adjusted probabilities and ARDs from mixed-effects logistic regression models with a random intercept for centre, fitted within 20 multiply imputed datasets (*n* = 490) and pooled using Rubin’s rules.

Models adjusted for age, baseline NIHSS score, ASPECTS, ASITN/SIR collateral grade, occlusion site, TOAST subtype, sex and admission glucose.

PT was modelled per 10 min (PT10) using restricted cubic splines with 4 knots at the 5th, 35th, 65th and 95th percentiles.

Probabilities were averaged over the observed covariate distribution with PT set to 60, 90, 120 or 150 min (fixed effects; centre random intercept set to 0).

ARDs are percentage-point differences compared with PT = 60 min.

### Mortality and the full 90-day disability spectrum

**Figure 3:**
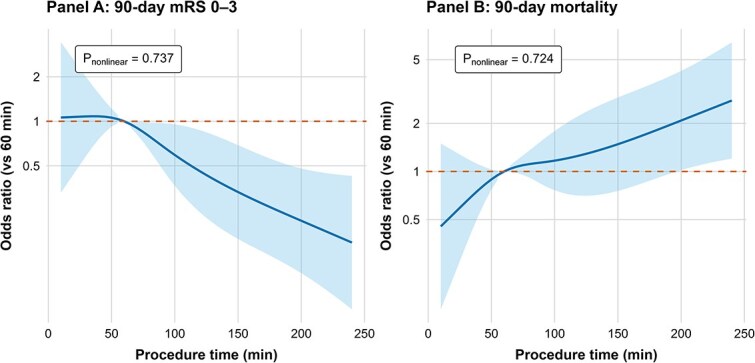
Restricted cubic spline analysis of the association between PT and 90-day outcomes. Adjusted ORs (solid line) with 95% CIs (shaded band) are shown across PT (minutes), referenced to PT = 60 min (dashed line, OR = 1). PT was modelled per 10 min using restricted cubic splines with knots at the 5th, 35th, 65th and 95th percentiles. *P* values for nonlinearity (insets) were obtained from Wald tests of the nonlinear spline components. Models were adjusted as in model 3 with centre as a random intercept. Longer PT was associated with lower odds of 90-day mRS 0–3 and higher odds of 90-day mortality, with no evidence of nonlinearity (*P* for nonlinear shown in insets). Panel A shows odds of 90-day mRS 0–3 (OR < 1 indicates lower odds of favourable outcome); panel B shows odds of 90-day mortality (OR > 1 indicates higher odds of death). Abbreviations: OR  =  odds ratio; PT  =  procedure time.

Ninety-day mortality rates were 36.8% (68/185), 44.7% (88/197) and 47.1% (48/102) across PT strata (Table 2). In model 3, extended PT was associated with higher mortality compared with PT ≤ 60 min (OR 2.51; 95% CI, 1.29–4.88; *P* = .007), with a monotonic trend across PT strata (*P* for trend = .007) (Table 2). Continuous modelling likewise showed higher mortality with longer PT (OR 1.07 per 10 min, 95% CI, 1.02–1.11; *P* = .002). Restricted cubic spline analyses showed no evidence of nonlinearity for mortality (*P* for nonlinearity = .724) (Figure 3). Marginally standardised mortality increased from 38.8% at 60 min to 45.2% at 150 min (ARD at 150 vs 60 min: +6.4 percentage points; 95% CI, −4.6 to +17.5) (Table 3).

Associations were directionally consistent across alternative functional thresholds (Table 2). For mRS 0–2, extended PT was associated with lower odds of an excellent outcome (model 3: OR 0.42; 95% CI, 0.20–0.88; *P* = .022; *P* for trend = .020). For mRS 0–4, extended PT was also associated with lower odds of a favourable outcome (model 3: OR 0.44; 95% CI, 0.23–0.84; *P* = .012; *P* for trend = .009). Analysis of the full 90-day mRS distribution demonstrated a shift towards worse disability with longer PT: median mRS was 4 (IQR, 2–6) for PT ≤ 60 min and 5 (IQR, 3–6) for both PT > 60–≤120 min and PT > 120 min (Table 2). In model 3 shift analyses, the odds of worse disability were higher for intermediate PT (OR 1.54, 95% CI, 1.03–2.31; *P* = .035) and extended PT (OR 2.36; 95% CI, 1.40–3.98; *P* = .001), with a monotonic trend (*P* for trend < .001) (Table 2). Continuous modelling similarly showed a worse mRS distribution with each 10-min increase in PT (model 3: OR 1.07; 95% CI, 1.04–1.11; *P* < .001).

### Robustness and safety analyses

Among patients achieving successful reperfusion (mTICI ≥ 2b), the association between longer PT and worse outcome persisted. Compared with PT ≤ 60 min, extended PT remained associated with lower odds of mRS 0–3 (OR 0.35; 95% CI, 0.16–0.74; *P* = .006), with corresponding results reported in Tables S6 and S7. Propensity score–weighted doubly robust analyses (Tables S2 and S3) and complete-case analyses in the overall cohort (Tables S4 and S5) were directionally consistent with the primary results.

Symptomatic ICH occurred in 11.4% (21/185), 14.2% (28/197) and 15.7% (16/102) of patients across PT strata (Table 2). In the primary adjusted analyses, no statistically significant association between PT and sICH was observed in categorical models (extended vs ≤ 60 min: OR 1.53; 95% CI, 0.71–3.27; *P* = .274; *P* for trend = .274) or in continuous modelling (OR 1.03 per 10 min; 95% CI, 0.98–1.08; *P* = .201). Complete-case analyses were similarly non-significant, whereas 1 weighted sensitivity analysis yielded a nominally significant estimate at prolonged PTs (Table S3). Taken together, no consistent association between PT and sICH was observed across analytic approaches, although a modest increase in risk at prolonged PTs cannot be excluded.

Additional exploratory analyses did not support redefining the original ≤ 60-min reference category after subdivision into PT < 30 min and PT 30–60 min (Tables S9 and S10). In an MI-based PT-by-collateral interaction analysis, no consistent effect modification was observed across functional outcomes, although a nominal interaction was found for mortality (*P* for interaction = .047) (Table S11).

## Discussion

In this nationwide multicentre cohort of imaging-defined large-core stroke undergoing EVT, longer PT was associated with progressively worse 90-day functional outcome, greater disability across the full mRS distribution and higher mortality. Beyond confirming the direction of this association, the present analysis characterises the dose–response shape of the PT–outcome association specifically in large-core EVT: the risk gradient extended across the clinically encountered range of PTs rather than showing an abrupt inflection point. By combining dose–response modelling with adjusted absolute-risk estimates, our study translates PT from a procedural metric into a clinically interpretable intraprocedural risk gradient for large-core EVT.

Prior studies in general EVT populations have linked longer PT with worse outcomes, with some reports using pragmatic benchmarks such as a “golden hour” and others describing a more gradual deterioration with increasing PT.^14^^,^^15^ However, these observations largely derive from mixed core-burden populations, in which differences in baseline tissue status, collateral reserve and salvage potential can complicate interpretation of procedure-time patterns. In large-core EVT, where salvage potential is more constrained and procedural prolongation may carry particular prognostic weight, the dose–response shape and clinical magnitude of the PT–outcome association require more specific characterisation. In this context, our findings are directionally consistent with the recent SELECT2 secondary analysis,^16^ while extending prior work by formally assessing nonlinearity and translating the association onto an adjusted absolute-risk scale, thereby making the PT–outcome gradient more clinically interpretable in a real-world multicentre large-core EVT cohort.

Clinically, the value of this dose–response analysis is not to define a time at which EVT should automatically stop. Rather, prolonged PT should function as an intraprocedural warning signal that prompts the treating team to reassess whether further attempts still offer a reasonable chance of meaningful reperfusion at an acceptable procedural risk. The magnitude of the gradient was clinically meaningful: each 10-min increase in PT was associated with lower odds of 90-day mRS 0–3 and higher odds of 90-day mortality (Table 2), and the model-adjusted probability of achieving mRS 0–3 in the overall large-core EVT cohort declined from 44.5% at 60 min to 32.2% at 120 min and 27.5% at 150 min (Table 3). In practice, as thrombectomy extends beyond the original ≤ 60-min reference range, elapsed time alone should not determine whether the procedure is continued or stopped. Instead, reassessment should incorporate angiographic progress, likelihood of meaningful reperfusion, collateral/tissue profile, technical feasibility, procedural complexity, haemodynamic stability and emerging procedural risks. The broader functional-outcome results support this interpretation: although the association with mRS 0–4 was less pronounced in the > 60–≤120-min category, extended PT was associated with lower odds of mRS 0–4, and analysis of the full 0–6 mRS distribution showed worse disability with longer PT. Because PT is defined only among patients undergoing EVT, these data cannot determine when EVT loses benefit relative to medical therapy; they quantify the increasing prognostic burden of prolonged procedures within an EVT-treated cohort.

These findings also clarify what PT represents in large-core EVT. Procedure time is unlikely to reflect procedural speed alone; rather, it integrates procedural efficiency, vascular and angiographic complexity, collateral/tissue profile, intraprocedural decision-making and continued ischaemic time-at-risk during attempted recanalisation.^2^^,^^27^ The additional exploratory analyses support this interpretation. Within the original ≤ 60-min reference category, patients with PT < 30 min had a more favourable collateral profile than those with PT 30–60 min, suggesting that very short PT may partly reflect more straightforward vascular and collateral conditions rather than technical efficiency alone (Tables S9 and S10). In the MI-based PT-by-collateral interaction analysis, formal tests did not show consistent effect modification across functional outcomes, although a nominal interaction was observed for mortality (Table S11). Thus, collateral status should help contextualise PT and support intraprocedural judgement, but it should not be used as a simple effect modifier, procedural stopping rule or substitute for real-time assessment.

Notably, the association between longer PT and worse outcome persisted among patients who achieved successful reperfusion (mTICI ≥ 2b; Tables S6 and S7), suggesting that the prognostic information carried by PT is not fully captured by final angiographic success alone. This subgroup analysis should nevertheless be interpreted cautiously, because successful reperfusion is itself a post-exposure procedural outcome related to procedure duration, case complexity and prognosis; restricting analyses to this subgroup may therefore introduce selection bias.^19^ Thus, this finding does not establish whether the association reflects direct harm from procedural prolongation, greater underlying procedural complexity, or both. We also observed no consistent association between PT and sICH across analytic approaches, although a modest increase in risk at prolonged PTs cannot be excluded. Future studies should further decompose PT into granular procedural segments—such as puncture-to-first device deployment, device-pass intervals, first-pass effect and timing of escalation to rescue strategies—to distinguish potentially modifiable delay from irreducible procedural complexity.

### Strengths

This study leveraged a prospective nationwide multicentre registry of 490 patients across 38 stroke centres, with standardised 90-day outcome assessment and core-laboratory adjudication of key imaging variables. The analysis included all patients with attempted EVT regardless of reperfusion status, and used prespecified confounder control with multiple imputation, IPTW with doubly robust estimation and complete-case sensitivity analyses. Dose–response modelling with restricted cubic splines and model-adjusted absolute-risk estimates at clinically relevant PT values improved the clinical interpretability of the findings.

### Limitations

Several limitations should be acknowledged. First, although the MAGIC registry was prospectively established, the present study was a retrospective observational analysis of prospectively collected data; despite prespecified confounder control and robustness analyses, residual confounding cannot be fully excluded, particularly from unmeasured anatomic difficulty, thrombus characteristics, operator-level decision-making and real-time decisions to continue or terminate thrombectomy. Second, PT is a composite procedural measure reflecting efficiency, access difficulty, recanalisation complexity, intraprocedural decision-making and ongoing ischaemic exposure; intraprocedural variables were not included in primary models because they may occur during or after the development of PT, which limits our ability to fully separate procedural duration from procedural management and technical difficulty. Third, the < 30 vs 30–60-min, PT-by-collateral and successful-reperfusion analyses were exploratory and should be interpreted as hypothesis-generating; in particular, analyses restricted to successful reperfusion should be interpreted cautiously, as conditioning on a post-exposure outcome may introduce selection bias. Fourth, large core was defined by noncontrast CT ASPECTS ≤ 5, which is pragmatic but not directly interchangeable with perfusion- or MRI-based definitions; the cohort was derived from stroke centres in China, procedural time recording may vary across sites and uncertainty was greater at the extremes of the PT distribution, which may limit generalisability.

## Conclusion

In this nationwide multicentre cohort of large-core stroke undergoing EVT, longer PT was consistently associated with worse 90-day functional outcome and higher mortality. By characterising the dose–response pattern and quantifying model-adjusted absolute risks, our findings show that the probability of achieving mRS 0–3 declined substantially across clinically encountered PTs, from 44.5% at 60 min to 27.5% at 150 min. These results support understanding PT as a continuous intraprocedural risk indicator that may inform ongoing procedural reassessment rather than as a simple procedural duration metric. Future studies should identify which components of PT—technical efficiency, anatomic complexity and intraprocedural decision-making—are most modifiable and whether optimising them improves outcomes in large-core EVT.

## Supplementary Material

supplement_aakag062

## Data Availability

The data that support the findings of this study are available from the corresponding author upon reasonable request.
