# Supplementary material for: Procedure time and 90-day outcomes after endovascular thrombectomy for large-core stroke
Source: Eur Stroke J. 2026 Jun 26;11(6):aakag062. doi: 10.1093/esj/aakag062 (PMC13308648; doi:10.1093/esj/aakag062)
Supplement: supplement_aakag062 [file supplement_aakag062.docx]

**Supplementary Materials**

**Content**

[**Supplementary Methods** 3](#_Toc228650613)

[**eMethods S1. Directed Acyclic Graph (DAG) and Covariate Selection** 3](#_Toc228650614)

[**eMethods S2. Missing Data Patterns and Multiple Imputation** 6](#_Toc228650615)

[**eMethods S3. Regression Models** 7](#_Toc228650616)

[**eMethods S4. Inverse Probability of Treatment Weighting and Doubly Robust Analysis (IPTW)** 9](#_Toc228650617)

[**eMethods S5. Restricted Cubic Spline Analysis** 11](#_Toc228650618)

[**eMethods S6. Complete-case Sensitivity Analyses and Assessment of Proportional Odds Assumption** 12](#_Toc228650619)

[**eMethods S7. Additional Exploratory Analyses** 15](#_Toc228650620)

[**Supplementary Tables** 17](#_Toc228650621)

[**eTable 1. Distribution and Proportion of Missing Data for Variables Used in Regression Models** 17](#_Toc228650622)

[**eTable 2. Baseline characteristics across procedure time categories before and after inverse probability of treatment weighting.** 19](#_Toc228650623)

[**eTable 3. Doubly robust inverse probability–weighted analysis of procedure time categories and 90-day outcomes** 23](#_Toc228650624)

[**eTable 4. Complete-case sensitivity analyses of categorical procedure time and 90-day outcomes** 25](#_Toc228650625)

[**eTable 5. Complete-case sensitivity analysis: association between continuous procedure time and 90-day outcomes** 29](#_Toc228650626)

[**eTable 6. Complete-case sensitivity analyses of categorical procedure time and outcomes in patients achieving successful reperfusion (mTICI ≥2b)** 32](#_Toc228650627)

[**eTable 7. Complete-case sensitivity analyses of continuous procedure time and outcomes in patients achieving successful reperfusion (mTICI ≥2b)** 36](#_Toc228650628)

[**eTable 8. Reference-patient–based adjusted probabilities and absolute risk differences at key procedure times for 90-day mRS 0–3 and mortality** 39](#_Toc228650629)

[eTable 9. Baseline and procedural characteristics among patients with procedure time ≤60 minutes, stratified by <30 versus 30–60 minutes. 40](#_Toc228650630)

[eTable 10. Exploratory outcome analysis among patients with procedure time ≤60 minutes, stratified by <30 versus 30–60 minutes. 42](#_Toc228650631)

[eTable 11. Exploratory PT-by-collateral interaction analysis in the overall EVT cohort. 43](#_Toc228650632)

[**Supplementary Figures** 45](#_Toc228650633)

[**eFigure S1. Directed Acyclic Graph (DAG) of the Causal Structure Between Procedure Time (PT) and 90-Day Outcomes.** 45](#_Toc228650634)

[**eFigure S2. Missing Data for Key Continuous Variables** 46](#_Toc228650635)

[**eFigure S3. Distribution Comparison of Observed vs. Imputed Values for Continuous Variables** 47](#_Toc228650636)

[**eFigure S4. Internal Diagnostic Plot for 20 Imputed Datasets** 48](#_Toc228650637)

[**eFigure S5. Adjusted predicted probabilities of 90-day outcomes across procedure time.** 49](#_Toc228650638)

[**eFigure S6. Covariate balance before and after inverse probability of treatment weighting across procedure time categories.** 50](#_Toc228650639)

# **Supplementary Methods**

## **eMethods S1. Directed Acyclic Graph (DAG) and Covariate Selection**

**S1.0 Definition of Procedure Time (PT)**

The primary exposure in this study was procedure time (PT), defined as the interval from femoral arterial puncture to the end of endovascular treatment. This definition applied to all patients who underwent attempted EVT (n=490), including those with successful reperfusion (mTICI ≥2b; n=423) and those with failed reperfusion (mTICI <2b; n=67). For patients with successful reperfusion, the “end of procedure” was defined as the time at which mTICI ≥2b was first achieved. For patients without successful reperfusion, the “end of procedure” was defined as the time of procedure termination or the final angiographic assessment. This definition is consistent with the original MAGIC report (Guo et al., 2024) and with commonly used definitions in the EVT literature.

Among the 490 patients, PT was recorded for 484 patients (missing in 6 patients). Median PT was 71 minutes (IQR, 51–115). Using prespecified cut points, PT was categorized as ≤60 minutes (n=185), >60 to ≤120 minutes (n=197), and >120 minutes (n=102).

**S1.1 DAG Construction and Key Pathways**

We used DAGitty to construct a directed acyclic graph with PT as the exposure and 90-day outcomes as the endpoints (eFigure 1). Major nodes in the DAG included:

1. Baseline stroke characteristics: baseline NIHSS, ASPECTS, collateral status (modified ASITN/SIR grade), occlusion site (intracranial internal carotid artery or middle cerebral artery M1/M2), and stroke etiology (TOAST classification).
2. Demographics and baseline status: age, sex, prestroke mRS, and conventional vascular risk factors (eg, hypertension, diabetes, atrial fibrillation, smoking, dyslipidemia).
3. Treatment and workflow variables: PT (exposure), thrombectomy technique and number of passes, anesthesia modality, intravenous thrombolysis (IVT), workflow time metrics (eg, onset-to-imaging time, onset-to-puncture time), and treating center.
4. Outcomes: 90-day functional outcome (mRS 0–3 vs 4–6) and 90-day all-cause mortality.

The DAG indicated that baseline severity and infarct extent (NIHSS, ASPECTS, collateral status), occlusion site, TOAST subtype, and age influence both PT and 90-day outcomes, forming the primary backdoor confounding paths. Thrombectomy technique, number of passes, and anesthesia modality were considered more likely to lie on the pathway from PT to outcomes (ie, potential mediators) and were therefore not included in the primary adjustment models. Center-level differences may reflect systematic variation in case mix and workflow organization and introduce clustering.

**S1.2 Minimally Sufficient Adjustment Set, Center Effects, and Expanded Adjustment Set**

Based on the backdoor criterion and clinical plausibility, we prespecified two covariate adjustment frameworks for outcome regression:

1. Model 1 (minimally sufficient adjustment set): age, baseline NIHSS, ASPECTS, collateral grade (modified ASITN/SIR), occlusion site, and TOAST subtype.
2. Model 2 (expanded adjustment set): Model 1 covariates plus sex and admission glucose.

Because this was a multicenter cohort, the center variable was used to reflect system-level differences across hospitals (eg, patient selection, workflow organization, and periprocedural management) and to account for clustering. Center effects were handled as follows: in unweighted outcome models using the multiple-imputation datasets (eMethods S3), center was adjusted using a random intercept; in IPTW-weighted outcome models (eMethods S4), center was included as fixed effects; in complete-case sensitivity analyses (eMethods S6), center was adjusted as fixed effects for the overall EVT cohort (eTable 4–5) and as random intercepts for the successful reperfusion subgroup (eTable 6–7).

**S1.3 Application of the Covariate Framework in Subsequent Analyses**

All subsequent statistical analyses were based on the covariate sets defined by the DAG framework:

1. Multiple imputation model: PT, Model 1 and Model 2 covariates, and additional fully observed variables that improve imputation performance were included as predictors in the MICE procedure (see eMethods S2).
2. Primary regression analyses (multiple-imputation datasets; unweighted): sequential models were fitted, including an unadjusted model, a Model 1–adjusted model, and a further adjusted model incorporating Model 2 covariates with a random intercept for center (corresponding to Models 1–3; see Tables 2 and 3).
3. IPTW analyses: within the multiple-imputation datasets, PT category (≤60, >60 to ≤120 min, >120 minutes) was used as the exposure, and generalized propensity scores were estimated using Model 2 covariates to construct IPTW. Weighted (ordinal) logistic regression models were then used to estimate associations between PT and outcomes in the weighted sample, with center included as fixed effects in the outcome models to control for between-center systematic differences, yielding doubly robust estimates.

## **eMethods S2. Missing Data Patterns and Multiple Imputation**

**S2.1 Extent and Patterns of Missing Data**

In this cohort, the primary exposure (PT and its 3-category variable) and outcome measures (90-day mRS, mortality, and symptomatic intracranial hemorrhage) were largely complete. Missingness was limited to 5 continuous variables: admission glucose (glucose; 12 missing), systolic blood pressure (SBP; 8 missing), diastolic blood pressure (DBP; 8 missing), PT (6 missing), and onset-to-puncture time (5 missing). All other covariates had no missing data (eFigure 2, eTable 1). eTable 1 summarizes missingness only for the exposure and covariates included in the regression models; although SBP, DBP, and onset-to-puncture time were not included in the primary regression models, they were incorporated as predictors in the imputation model, and their missingness distributions are shown in eFigure 2.

Missing cases did not appear to cluster within specific centers or within specific PT strata. Based on variable definitions and the data collection process, we considered missingness more likely to be related to observed baseline characteristics and workflow time metrics; therefore, multiple imputation was performed under a missing-at-random (MAR) assumption.

**S2.2 Implementation of Multiple Imputation**

Under the MAR assumption, we used multiple imputation by chained equations (MICE) to address missingness in the continuous variables described above:

1. Continuous variables were imputed using predictive mean matching (PMM).
2. Twenty imputed datasets were generated, with 20 iterations per chain.
3. The imputation model included PT, Model 1 and Model 2 covariates, and the center variable. In addition, the 5 variables with missingness served as predictors for one another within the chained-equations procedure.

Primary and secondary outcomes had no missingness and were included in the imputation model only as predictors (auxiliary variables), not as targets for imputation. All subsequent regression and IPTW analyses were performed separately within each of the 20 imputed datasets, and estimates and standard errors were pooled using Rubin’s rules.

**S2.3 Diagnostics and Internal Validation of Imputation**

We evaluated the adequacy of the imputation procedure using the following approaches:

1. Distributional comparisons (eFigure 3): we compared the distributions of glucose, SBP, DBP, PT, and onset-to-puncture time between observed and imputed data.
2. Internal validation (eFigure 4): for each variable with missingness, we temporarily set a subset of observed values to missing, imputed them using the prespecified MICE model, and compared imputed values against the true observed values to assess the direction and stability of prediction errors.

Based on these diagnostics, we considered the multiple-imputation specification acceptable in terms of missingness extent and distributional plausibility, and suitable to support subsequent regression modeling and IPTW–doubly robust analyses.

## **eMethods S3. Regression Models**

**S3.1 Overall Analysis Strategy**Regression models were fitted in the 20 datasets generated by multiple imputation using chained equations, and the regression coefficients and standard errors were combined using Rubin’s rules. Effect sizes are presented as odds ratios (OR) with 95% confidence intervals (CI), and P-values were calculated using two-sided tests.

**S3.2 Exposure Variable Specification**
The primary exposure variable was procedure time (PT), which was modeled in three forms according to the predefined scheme:

1. Continuous variable PT (increase by 1 minute)
2. Continuous variable PT10 (increase by 10 minutes)
3. Three-category PT (≤60, >60 to ≤120 min, >120 minutes, with ≤60 minutes as the reference).

**S3.3 Covariate Adjustment Framework**
The covariate set and stratified adjustment strategy were predefined based on eMethod 1 (S1.3), with models sequentially constructed as Crude, Model 1, Model 2, and Model 3, which additionally includes center effects. Unless otherwise specified, Models 1/2 refer to the individual-level covariate sets as described in S1.3.

**S3.4 Center Effect Handling**
As a multicenter study, patients were nested within different centers. To account for baseline risk differences and patient clustering across centers, center was incorporated as a random intercept in fully adjusted regression models, fitted using mixed-effects logistic regression (Model 3).

**S3.5 Binary Outcome Regression Models**
Binary outcomes included 90-day functional outcomes (mRS 0–3 vs 4–6; mRS 0–2 vs 3–6; mRS 0–4 vs 5–6), 90-day all-cause mortality, and symptomatic intracranial hemorrhage (sICH). For each outcome and each form of PT, we sequentially fitted Crude, Model 1, Model 2, and added center random intercept in Model 3.
The OR for continuous PT/PT10 is interpreted as the change in the odds of the outcome per 1-minute or 10-minute increase in PT; for three-category PT, ORs and 95% CI are reported for >60 to ≤120 minutes and >120 minutes compared to ≤60 minutes.

**S3.6 90-day mRS 0–6 Shift Analysis**
The 90-day mRS 0–6 was treated as an ordinal outcome and analyzed using shift analysis with the cumulative logit proportional-odds model. The same stratified adjustment strategy as for binary outcomes was used, sequentially constructing Crude, Model 1, Model 2, and including center random intercept in Model 3. ORs are interpreted as the cumulative odds ratio for moving toward a higher (worse) mRS category with an increase in PT, holding other variables constant.

**S3.7 Trend Test**
To evaluate the linear trend of the three-category PT, we sequentially coded the time strata as 1 (≤60 min), 2 (>60 to ≤120 min), 3 (>120 min), and refitted the model with this ordinal variable in a fully adjusted framework. The Wald test P-value was extracted for P for trend.

**S3.8 Statistical Software**
The following R packages were used: mice for multiple imputation, lme4 for mixed-effects logistic regression, ordinal for cumulative logit mixed-effects models, and broom.mixed for model summarization.

## **eMethods S4. Inverse Probability of Treatment Weighting and Doubly Robust Analysis (IPTW)**

**S4.1 Analysis Objective and Exposure Specification**To further assess the robustness of primary outcomes with respect to confounding control strategies, we conducted inverse probability of treatment weighting (IPTW) analysis across multiple imputed datasets. The exposure was defined as the pre-specified PT three-category variable (≤60, >60 to ≤120 min, >120 minutes; ≤60 minutes as the reference).

**S4.2 Generalized Propensity Score Estimation and Weight Construction**
Generalized propensity scores for the three-category procedure time were estimated within each imputed dataset using multinomial logistic regression with Model 2 covariates. ATE inverse probability weights (1/PS) were constructed and truncated at the 1st and 99th percentiles. Doubly robust analyses used these weights with additional adjustment for Model 2 covariates and center fixed effects. Estimates were pooled across imputations using Rubin’s rules.

**S4.3 Covariate Balance Before and After Weighting**
Standardized mean differences (SMD) were used to assess covariate balance before and after weighting, and Love plots were employed for visual representation.

**S4.4 Weighted Outcome Models and Doubly Robust Estimation**
We assessed the association between PT categories and outcomes in the weighted sample:

1. For binary outcomes (mRS 0–3, mRS 0–2, mRS 0–4, 90-day all-cause mortality, and symptomatic intracranial hemorrhage), weighted logistic regression was used.
2. For ordinal outcomes (90-day mRS 0–6), weighted proportional odds (cumulative logit) models were used for shift analysis.

To obtain doubly robust estimates, the above weighted outcome models were further adjusted for the same Model 2 covariates. Center was included as fixed effects in outcome models to control for systematic between-center differences.

**S4.5 Combining Rules and Results Presentation**
After propensity scores, weight construction, balance assessment, and outcome modeling were completed in each imputed dataset, Rubin’s rules were applied to combine regression coefficients and standard errors. Results are presented as odds ratios (OR) with 95% confidence intervals (CI), and P-values were calculated using two-sided tests.

## **eMethods S5. Restricted Cubic Spline Analysis**

**S5.1 Analysis Objective**To evaluate potential nonlinearity in the association between procedure time (PT) and 90-day outcomes, PT was modeled per 10-minute increment (PT10) using restricted cubic splines (RCS) in mixed-effects logistic regression models fitted within the multiply imputed datasets.

**S5.2 Knot Placement and Reference Value**RCS models used four internal knots placed at the 5th, 35th, 65th, and 95th percentiles of the PT10 distribution. The reference value was set at **PT = 60 minutes (PT10 = 6)**, and effect estimates were expressed relative to this reference (odds ratio = 1). Knot locations were determined from the PT10 distribution in the first completed dataset.

**S5.3 Regression Models and Covariate Adjustment**RCS analyses were conducted for binary outcomes, including 90-day mRS 0–3 and 90-day all-cause mortality. For each imputed dataset, a mixed-effects logistic regression model was fitted with **center as a random intercept**. Models were adjusted for the prespecified covariates used in the primary analysis: age, baseline NIHSS score, ASPECTS, modified ASITN/SIR collateral grade, occlusion site, TOAST subtype, sex, and admission glucose.

**S5.4 Pooling Across Imputations and Curve Construction**

Models were fitted in each of the 20 imputed datasets. For the odds ratio RCS curves, log-odds contrasts at each PT value relative to PT = 60 minutes were computed within each imputed dataset and then pooled pointwise using Rubin’s rules to obtain combined estimates and 95% confidence intervals.

To facilitate clinical interpretation, adjusted predicted probability curves were additionally generated from the fitted spline models for a reference patient (continuous covariates fixed at the median and categorical covariates fixed at the modal category), varying PT over a prespecified grid; predicted probabilities and uncertainty were pooled across imputations using Rubin’s rules.

**S5.5 Nonlinearity Testing and Results Presentation**Nonlinearity was evaluated by conducting a joint Wald test on the nonlinear terms of the spline. Results were presented as RCS curves showing the trend of OR and 95% CI across varying levels of PT10, with PT=60 minutes (PT10=6) as the reference (OR=1).

## **eMethods S6. Complete-case Sensitivity Analyses and Assessment of Proportional Odds Assumption**

Complete-case (CC) sensitivity analyses were performed to assess robustness of the main findings under a complete-data framework. All exposure definitions, outcomes, covariate sets (Models 1–3), center-effect specification, and interpretation of odds ratios followed the prespecified methods described in eMethods S1–S3.

**S6.1. CC cohort construction and missing-data handling**

CC analyses were conducted in two analytic populations:
(A) Overall EVT population (CC).
This CC analysis included all patients who underwent attempted EVT and met the following criteria:
1. Observed PT (i.e., non-missing procedure time); and
2. Complete covariate data required for the fully adjusted fixed-effects set (Model 2 covariates). Patients failing either criterion were excluded from CC regression datasets. Results are reported in eTable 4 (categorical PT) and eTable 5 (continuous PT).
(B) Successful reperfusion population (CC; mTICI ≥2b).
This CC analysis was restricted to patients achieving successful reperfusion (mTICI ≥2b) and additionally required:
1. Observed PT; and
2. Complete Model 2 covariates.
Therefore, the CC sample size in this analysis may be smaller than the total number of successfully reperfused patients because exclusions can arise from missing PT and/or missing covariates. Results are reported in eTable 6 (categorical PT) and eTable 7 (continuous PT).

**S6.2. Exposure parameterizations used in CC analyses**

Consistent with eMethods S3, PT was modeled as:
1. Categorical PT (≤60, >60 to ≤120, and >120 minutes; reference = ≤60); and
2. Continuous PT modeled per 10-minute increase and per 1-minute increase.
For categorical PT analyses, P for trend was obtained using a Wald test by modeling the PT categories as an ordinal variable coded 1, 2, and 3, while keeping the same fixed- and random-effects structure as Model 3 (eMethods S3.7).

**S6.3. Center effects and model fitting in CC analyses**

Model covariates followed eMethods S3. Crude, Model 1, and Model 2 were fitted without adjustment for center. Model 3 center specification differed between populations:
**Overall EVT complete-case population (eTable 4–5):** Center was adjusted as fixed effects.
1. For binary outcomes, logistic regression was used.
2. For ordinal 90-day mRS (0–6), proportional-odds models were used.

**Successful reperfusion complete-case population (eTable 6–7):** Center was adjusted using a random intercept.
1. For binary outcomes, mixed-effects logistic regression was used (lme4::glmer).
2. For ordinal 90-day mRS (0–6), mixed-effects proportional-odds models were used (ordinal::clmm)."

**S6.4. Reporting denominators for descriptive outcome frequencies in CC tables**

Among patients with successful reperfusion (n=423), procedure time (PT) was observed in 417 patients (6 missing). Descriptive statistics and outcome counts were summarized in this cohort (n=417). For regression analyses, complete cases were required for covariates included in the adjusted models; because admission glucose was included in Model 2/Model 3, nine patients with missing glucose were excluded, yielding an analytic sample of n=408.
For the ordinal mRS (0–6), descriptive results were summarized as median (IQR) by PT group, and effect estimates were derived from proportional-odds (shift) models (as specified in eMethods S3.6).

**S6.5 Assessment of the proportional-odds assumption (Brant test)**

For the ordinal 90-day mRS (0–6) shift analyses, proportional-odds (PO) models assume that the exposure effect is constant across cumulative logit cut points. We evaluated this PO assumption in the **overall EVT complete-case dataset** using the Brant test. Because including center as fixed effects in a PO assumption test may result in over-parameterization given the large number of centers, we conducted the Brant test based on the Model 2 specification without the center term, using PT per 10-minute increase as the exposure and adjusting for the individual-level Model 2 covariates (age, baseline NIHSS, ASPECTS, modified ASITN/SIR collateral grade, occlusion site, TOAST subtype, sex, and admission glucose). The global test did not indicate violation of the proportional-odds assumption (Omnibus χ²=56.263, df=60, P=0.613).

**S6.6 Cumulative link mixed model (CLMM) and center-level heterogeneity**

To further account for clustering by center within a random-effects framework and to quantify between-center heterogeneity for the ordinal mRS shift outcome, we additionally fitted a cumulative link mixed model (CLMM; logit link) in the overall EVT complete-case dataset, specifying center as a random intercept and including the Model 2 covariates as fixed effects. This approach provides fixed-effects ORs with 95% CIs while simultaneously estimating the variance of the center-specific random intercept. The intraclass correlation coefficient (ICC) was derived from the random-intercept variance under a logistic mixed-model approximation (using the logistic distribution variance π²/3) to summarize the proportion of outcome variability attributable to between-center differences.

## **eMethods S7. Additional Exploratory Analyses**

To assess potential heterogeneity within the original ≤60-minute PT reference category, we performed an exploratory analysis restricted to patients with observed PT ≤60 minutes. Patients were subdivided into PT <30 minutes and PT 30–60 minutes, with PT <30 minutes as the reference group. Baseline and procedural characteristics were summarized descriptively. Exploratory outcome models were fitted for ordinal 90-day mRS shift, mRS 0–2, mRS 0–3, mRS 0–4, mortality, and sICH. Crude models included only the PT subgroup variable. Model 1 adjusted for age, baseline NIHSS score, ASPECTS, ASITN/SIR collateral grade, occlusion site, and TOAST subtype. Model 2 additionally adjusted for sex and admission glucose.

To evaluate whether collateral status modified the association between PT and outcomes, we performed an exploratory PT-by-collateral interaction analysis in the overall EVT cohort. Collateral status was dichotomized as poor collaterals (ASITN/SIR 0–1) versus better collaterals (ASITN/SIR 2–4, including intermediate and good collateral status). PT was modeled continuously per 10-minute increase. Models included PT10, collateral group, the PT10-by-collateral interaction term, age, baseline NIHSS score, ASPECTS, occlusion site, TOAST subtype, sex, and admission glucose. Models were fitted separately in each of the 20 imputed datasets and pooled using Rubin’s rules. P for interaction tested whether the PT–outcome association differed between poor and better collateral strata.

# **Supplementary Tables**

**eTable 1. Distribution and Proportion of Missing Data for Variables Used in Regression Models**

| Variable | | N total | N missing | % missing |
| --- | --- | --- | --- | --- |
| **Exposure** | |  |  |  |
|  | Procedure time (PT) | 490 | 6 | 1.2 |
| **Outcome** | |  |  |  |
|  | 90-day mRS score (0–6) | 490 | 0 | 0 |
|  | mRS 0-3 | 490 | 0 | 0 |
|  | mRS 0-2 | 490 | 0 | 0 |
|  | mRS 0-4 | 490 | 0 | 0 |
|  | Death | 490 | 0 | 0 |
|  | sICH | 490 | 0 | 0 |
| **Covariates (Model 1)** | |  |  |  |
|  | Age | 490 | 0 | 0 |
|  | Baseline NIHSS | 490 | 0 | 0 |
|  | ASPECTS | 490 | 0 | 0 |
|  | ASITN/SIR collateral grade | 490 | 0 | 0 |
|  | Occlusion site | 490 | 0 | 0 |
|  | TOAST classification | 490 | 0 | 0 |
| **Covariates (Model 2)** | |  |  |  |
|  | glucose | 490 | 12 | 2.4 |
|  | sex | 490 | 0 | 0 |
| **Center** | | 490 | 0 | 0 |
| Notes: Data show the number and percentage of missing values for each variable used in the regression analyses (exposure, outcomes, and covariates).  The 90-day mRS (0–6) was complete; binary functional outcomes derived from mRS therefore had no missing values.  Missing values in PT and glucose were handled using multiple imputation by chained equations (20 datasets; see eMethods S2).  Abbreviations: PT, procedure time; mRS, modified Rankin Scale; NIHSS, National Institutes of Health Stroke Scale; ASPECTS, Alberta Stroke Program Early CT Score; ASITN/SIR, American Society of Interventional and Therapeutic Neuroradiology/Society of Interventional Radiology collateral grading system; TOAST, Trial of Org 10172 in Acute Stroke Treatment classification; sICH, symptomatic intracranial hemorrhage. | | | | |

**eTable 2. Baseline characteristics across procedure time categories before and after inverse probability of treatment weighting.**

|  | | Before IPTW | | | After IPTW | | |
| --- | --- | --- | --- | --- | --- | --- | --- |
|  | | Fast group | Intermediate group | Extended group | Fast group | Intermediate group | Extended group |
| Age, y, median (IQR) | | 69.00 (59.00, 78.00) | 69.00 (60.00, 77.00) | 71.00 (57.00, 78.00) | 68.00 (59.00, 78.00) | 68.00 (59.00, 77.00) | 72.00 (58.00, 79.00) |
| Sex, male, n (%) | | 110 (58.5) | 110 (55.3) | 61 (59.2) | 278 (57.2) | 280 (57.1) | 276 (56.9) |
| Glucose | | 7.00 (5.80, 8.63) | 7.50 (6.20, 9.05) | 7.05 (5.93, 9.13) | 7.10 (5.87, 9.09) | 7.25 (6.10, 8.80) | 7.01 (5.95, 9.42) |
| Blood pressure, mm Hg, median (IQR) | |  |  |  |  |  |  |
|  | Systolic | 140.00 (128.00, 159.00) | 150.00 (134.00, 170.00) | 143.00 (123.00, 160.00) | 145.00 (129.00, 160.00) | 150.00 (133.00, 170.00) | 143.00 (123.00, 160.00) |
|  | Diastolic | 85.00 (76.00, 96.00) | 87.00 (76.00, 98.00) | 84.00 (75.00, 92.00) | 86.00 (76.00, 96.00) | 87.00 (76.00, 98.00) | 82.00 (74.00, 92.00) |
| Medical history, n (%) | |  |  |  |  |  |  |
|  | Hypertension | 115 (61.2) | 119 (59.8) | 63 (61.2) | 298 (61.4) | 285 (58.2) | 284 (58.6) |
|  | Hyperlipidemia | 46 (24.5) | 40 (20.1) | 20 (19.4) | 117 (24.0) | 102 (20.9) | 89 (18.4) |
|  | Diabetes | 30 (16.0) | 31 (15.6) | 12 (11.7) | 90 (18.5) | 68 (13.9) | 57 (11.7) |
|  | Smoking | 63 (33.5) | 57 (28.6) | 31 (30.1) | 161 (33.0) | 146 (29.8) | 139 (28.8) |
|  | Atrial fibrillation | 95 (50.5) | 86 (43.2) | 40 (38.8) | 221 (45.4) | 210 (42.9) | 237 (48.9) |
| ASPECTS, median (IQR) | | 4.00 (2.00, 5.00) | 4.00 (2.00, 5.00) | 4.00 (3.00, 5.00) | 4.00 (2.00, 5.00) | 4.00 (2.00, 5.00) | 4.00 (3.00, 5.00) |
| NIHSS score, median (IQR) | | 17.00 (14.00, 20.00) | 17.00 (14.00, 20.00) | 16.00 (13.00, 21.00) | 17.00 (14.00, 20.00) | 17.00 (14.00, 20.00) | 16.00 (13.00, 21.00) |
| ASITN/SIR, n (%) | |  |  |  |  |  |  |
|  | 0–1 | 96 (51.1) | 99 (49.7) | 44 (42.7) | 239 (49.1) | 234 (47.9) | 257 (53.0) |
|  | 2 | 62 (33.0) | 67 (33.7) | 40 (38.8) | 168 (34.5) | 170 (34.8) | 163 (33.5) |
|  | 3-4 | 30 (16.0) | 33 (16.6) | 19 (18.4) | 80 (16.5) | 85 (17.3) | 65 (13.5) |
| TOAST, n (%) | |  |  |  |  |  |  |
|  | LAA | 40 (21.3) | 65 (32.7) | 41 (39.8) | 137 (28.1) | 147 (30.1) | 140 (28.9) |
|  | CE | 126 (67.0) | 111 (55.8) | 40 (38.8) | 281 (57.8) | 276 (56.4) | 279 (57.4) |
|  | Other/unknown | 22 (11.7) | 23 (11.6) | 22 (21.4) | 68 (14.0) | 66 (13.5) | 66 (13.6) |
| Occlusion site, n (%) | |  |  |  |  |  |  |
|  | ICA | 70 (37.2) | 88 (44.2) | 48 (46.6) | 202 (41.6) | 202 (41.3) | 217 (44.8) |
|  | M1 segment | 102 (54.3) | 94 (47.2) | 37 (35.9) | 237 (48.8) | 237 (48.5) | 222 (45.8) |
|  | M2 segment | 16 (8.5) | 17 (8.5) | 18 (17.5) | 47 (9.6) | 50 (10.2) | 46 (9.4) |
| First choice of EVT, n (%) | |  |  |  |  |  |  |
|  | Stent thrombectomy | 62 (33.0) | 75 (37.7) | 42 (40.8) | 157 (32.4) | 187 (38.1) | 200 (41.2) |
|  | Aspiration | 112 (59.6) | 108 (54.3) | 51 (49.5) | 285 (58.5) | 264 (53.9) | 251 (51.8) |
|  | Others | 14 (7.4) | 16 (8.0) | 10 (9.7) | 44 (9.1) | 39 (8.0) | 34 (7.0) |
| IVT, n (%) | | 43 (22.9) | 48 (24.1) | 31 (30.1) | 116 (23.9) | 121 (24.7) | 130 (26.7) |
| OTI, min, median (IQR) | | 278.00 (170.00, 398.00) | 317.00 (175.00, 523.00) | 227.00 (107.00, 410.00) | 271.00 (170.00, 398.00) | 320.00 (175.00, 523.00) | 282.00 (149.00, 410.00) |
| OTP, min, median (IQR) | | 360.00 (232.00, 500.00) | 380.00 (256.00, 610.00) | 351.00 (197.00, 530.00) | 360.00 (230.00, 500.00) | 380.00 (256.00, 610.00) | 352.00 (224.00, 530.00) |
| General anesthesia, n (%) | | 41 (21.8) | 20 (10.1) | 24 (23.3) | 108 (22.2) | 48 (9.9) | 96 (19.8) |
| Notes: “Before IPTW” summarizes the first imputed dataset (N=490) within each procedure-time category (see eMethods S2 and S4). Values are median (interquartile range) for continuous variables and No. (%) for categorical variables in the unweighted sample.  Procedure time (PT) categories were defined as fast (≤60 min), intermediate (>60 to ≤120 min), and extended (>120 min).  “After IPTW” summarizes the weighted pseudo-population for the average treatment effect (ATE) after inverse probability of treatment weighting; percentages are weighted. Weights were truncated at the 1st and 99th percentiles as described in eMethods S4. Abbreviations: ATE, average treatment effect; ASPECTS, Alberta Stroke Program Early CT Score; ASITN/SIR, American Society of Interventional and Therapeutic Neuroradiology/Society of Interventional Radiology collateral grading system; CE, cardioembolism; EVT, endovascular thrombectomy; ICA, internal carotid artery; IQR, interquartile range; IVT, intravenous thrombolysis; LAA, large-artery atherosclerosis; NIHSS, National Institutes of Health Stroke Scale; OTI, onset-to-imaging time; OTP, onset-to-puncture time; PT, procedure time; IPTW, inverse probability of treatment weighting; sICH, symptomatic intracranial hemorrhage. | | | | | | | |

**eTable 3. Doubly robust inverse probability–weighted analysis of procedure time categories and 90-day outcomes**

| Outcome | | 60-120 min vs ≤60 min | | >120 min vs ≤60 min | | P for trend |
| --- | --- | --- | --- | --- | --- | --- |
|  | | **OR (95% CI)** | P value | **OR (95% CI)** | P value |  |
| Primary outcome | |  |  |  |  |  |
|  | mRS 0-3 | 0.43 (0.30–0.62) | <0.001 | 0.33 (0.21–0.50) | <0.001 | <0.001 |
| Secondary outcome | |  |  |  |  |  |
|  | mRS 0-2 | 0.70 (0.47–1.04) | 0.079 | 0.33 (0.21–0.52) | <0.001 | <0.001 |
|  | mRS 0-4 | 0.61 (0.42–0.87) | 0.007 | 0.41 (0.28–0.61) | <0.001 | <0.001 |
| Ordinal outcome | |  |  |  |  |  |
|  | mRS 0–6 | 1.59 (1.22–2.09) | 0.001 | 2.37 (1.75–3.14) | <0.001 | <0.001 |
| Safety outcome | |  |  |  |  |  |
|  | Mortality | 1.66 (1.16–2.37) | 0.005 | 2.51 (1.71–3.67) | <0.001 | <0.001 |
|  | sICH | 1.37 (0.86–2.17) | 0.182 | 1.85 (1.12–3.05) | 0.016 | 0.015 |
| Notes: Odds ratios (ORs) and 95% confidence intervals (CIs) were obtained from multiple-imputation–based inverse probability of treatment weighting (IPTW) models with additional covariate adjustment (doubly robust specification). Binary outcomes (90-day mRS 0–3, 0–2, 0–4, 90-day Mortality, and symptomatic intracranial hemorrhage [sICH]) were analyzed using weighted logistic regression, and the ordinal 90-day mRS (0–6) was analyzed using a weighted proportional-odds model. Procedure time (PT) was categorized as ≤60, >60 to ≤120 min, and >120 minutes, with ≤60 minutes as the reference.  IPTW used ATE weights (1/PS) truncated at the 1st/99th percentiles; doubly robust outcome models additionally adjusted for Model 2 covariates and center fixed effects; results were pooled across imputations using Rubin’s rules. P for trend was derived by modeling PT category as an ordinal variable to test for a linear trend across the three PT categories within the same weighted models. Estimates were combined across 20 imputed datasets using Rubin’s rules. Abbreviations: ATE, average treatment effect; CI, confidence interval; IPTW, inverse probability of treatment weighting; mRS, modified Rankin Scale; OR, odds ratio; PT, procedure time; sICH, symptomatic intracranial hemorrhage. | | | | | | |

**eTable 4. Complete-case sensitivity analyses of categorical procedure time and 90-day outcomes**

| Outcome | | No (%) | Crude OR | p value | Model 1 | | Model 2 | | Model 3 | |
| --- | --- | --- | --- | --- | --- | --- | --- | --- | --- | --- |
|  |  |  |  |  | Adjusted OR | p value | Adjusted OR | p value | Adjusted OR | p value |
| Primary outcome | |  |  |  |  |  |  |  |  |  |
| mRS 0-3 | |  |  |  |  |  |  |  |  |  |
|  | ≤ 60 min | 83/179 (46.4) | Reference | — | Reference | — | Reference | — | Reference | — |
|  | 60-120 min | 62/191 (32.5) | 0.58 (0.38, 0.87) | 0.010 | 0.47 (0.29, 0.78) | 0.003 | 0.45 (0.27, 0.76) | 0.003 | 0.45 (0.26, 0.76) | 0.003 |
|  | >120 min | 32/102 (31.4) | 0.56 (0.34, 0.93) | 0.026 | 0.35 (0.18, 0.65) | 0.001 | 0.30 (0.15, 0.58) | <0.001 | 0.28 (0.14, 0.57) | <0.001 |
| Secondary outcome | | |  |  |  |  |  |  |  |  |
| mRS 0-2 | |  |  |  |  |  |  |  |  |  |
|  | ≤ 60 min | 49/179 (27.4) | Reference | — | Reference | — | Reference | — | Reference | — |
|  | 60-120 min | 37/191 (19.4) | 0.66 (0.41, 1.07) | 0.095 | 0.67 (0.40, 1.14) | 0.140 | 0.67 (0.39, 1.15) | 0.148 | 0.67 (0.39, 1.16) | 0.153 |
|  | >120 min | 16/102 (15.7) | 0.52 (0.27, 0.95) | 0.038 | 0.45 (0.21, 0.89) | 0.027 | 0.42 (0.20, 0.85) | 0.018 | 0.40 (0.19, 0.86) | 0.018 |
| mRS 0-4 | |  |  |  |  |  |  |  |  |  |
|  | ≤ 60 min | 103/179 (57.5) | Reference | — | Reference | — | Reference | — | Reference | — |
|  | 60-120 min | 91/191 (47.6) | 0.68 (0.46, 1.02) | 0.063 | 0.60 (0.37, 0.97) | 0.040 | 0.62 (0.37, 1.03) | 0.067 | 0.62 (0.37, 1.03) | 0.067 |
|  | >120 min | 48/102 (47.1) | 0.69 (0.43, 1.12) | 0.138 | 0.46 (0.24, 0.85) | 0.014 | 0.41 (0.21, 0.78) | 0.007 | 0.41 (0.22, 0.78) | 0.007 |
| mRS 0 to 6, Median (IQR) | |  |  |  |  |  |  |  |  |  |
|  | ≤ 60 min | 4 (2-6) | Reference | — | Reference | — | Reference | — | Reference | — |
|  | 60-120 min | 5 (3-6) | 1.55 (1.08, 2.24) | 0.018 | 1.59 (1.08, 2.34) | 0.019 | 1.60 (1.08, 2.38) | 0.019 | 1.56 (1.03, 2.36) | 0.035 |
|  | >120 min | 5 (3-6) | 1.68 (1.08, 2.61) | 0.022 | 2.19 (1.35, 3.60) | 0.002 | 2.45 (1.50, 4.07) | <0.001 | 2.52 (1.47, 4.36) | <0.001 |
| Mortality | |  |  |  |  | |  |  |  |  |
|  | ≤ 60 min | 63/179 (35.2) | Reference | — | Reference | — | Reference | — | Reference | — |
|  | 60-120 min | 84/191 (44.0) | 1.39 (0.92, 2.10) | 0.116 | 1.55 (0.96, 2.51) | 0.077 | 1.51 (0.91, 2.52) | 0.112 | 1.52 (0.91, 2.57) | 0.113 |
|  | >120 min | 48/102 (47.1) | 1.53 (0.94, 2.50) | 0.089 | 2.31 (1.26, 4.27) | 0.007 | 2.63 (1.40, 5.00) | 0.003 | 2.66 (1.39, 5.12) | 0.003 |
| sICH | |  |  |  |  |  |  |  |  |  |
|  | ≤ 60 min | 20/179 (11.2) | Reference | — | Reference | — | Reference | — | Reference | — |
|  | 60-120 min | 25/191 (13.1) | 1.29 (0.71, 2.39) | 0.404 | 1.33 (0.71, 2.51) | 0.379 | 1.09 (0.56, 2.13) | 0.801 | 1.09 (0.56, 2.11) | 0.801 |
|  | >120 min | 16/102 (15.7) | 1.45 (0.71, 2.92) | 0.296 | 1.49 (0.69, 3.14) | 0.299 | 1.57 (0.72, 3.39) | 0.248 | 1.57 (0.73, 3.39) | 0.248 |
| Note: Complete-case analysis included 472 patients with complete data (18 patients with missing values were excluded from the total cohort of 490).  Data are odds ratios (ORs) with 95% confidence intervals (CIs) derived from logistic regression models for binary outcomes and proportional-odds models for the ordinal outcome.  Procedure time was categorized as ≤60, >60 to ≤120 min, and >120 minutes; the ≤60-minute group served as the reference category (OR=1.00).  “Crude” denotes unadjusted models.  Model 1 was adjusted for age, baseline NIHSS score, ASPECTS, ASITN/SIR collateral grade, occlusion site, and TOAST subtype.  Model 2 was additionally adjusted for sex and admission glucose.  Model 3 was additionally adjusted for center as fixed effects.  “No./Total (%)” indicates the number and percentage of patients with the outcome within each procedure-time category for binary outcomes; for the ordinal 90-day mRS (0–6) outcome, values are presented as median (IQR) by procedure-time category.  The proportional-odds assumption was assessed using the Brant test in the complete-case dataset (see eMethods S6).  For dichotomous functional outcomes (mRS 0–2, 0–3, and 0–4), the event was defined as a favourable outcome; therefore, ORs <1 indicate lower odds of achieving a favourable outcome with longer procedure time.  For the ordinal mRS 0–6 outcome, ORs >1 indicate a shift towards higher (worse) mRS categories with longer procedure time.  Abbreviations: mRS, modified Rankin Scale; sICH, symptomatic intracranial hemorrhage; OR, odds ratio; CI, confidence interval; NIHSS, National Institutes of Health Stroke Scale; ASPECTS, Alberta Stroke Program Early CT Score; ASITN/SIR, American Society of Interventional and Therapeutic Neuroradiology/Society of Interventional Radiology collateral grading system; TOAST, Trial of Org 10172 in Acute Stroke Treatment classification. | | | | | | | | | | |

**eTable 5. Complete-case sensitivity analysis: association between continuous procedure time and 90-day outcomes**

| Outcome | | Crude OR | p value | Model 1 | | Model 2 | | Model 3 | |
| --- | --- | --- | --- | --- | --- | --- | --- | --- | --- |
|  |  |  |  | Adjusted OR | p value | Adjusted OR | p value | Adjusted OR | p value |
| Primary outcome | |  |  |  |  |  |  |  |  |
| mRS 0-3 | |  |  |  |  |  |  |  |  |
|  | Per 10 min | 0.95 (0.91, 0.98) | 0.005 | 0.91 (0.87, 0.96) | <0.001 | 0.90 (0.86, 0.95) | <0.001 | 0.90 (0.85, 0.95) | <0.001 |
|  | Per 1 min | 0.99 (0.99, 1.00) | 0.005 | 0.99 (0.99, 1.00) | <0.001 | 0.99 (0.98, 0.99) | <0.001 | 0.99 (0.98, 0.99) | <0.001 |
| Secondary outcome | |  |  |  |  |  |  |  |  |
| mRS 0-2 | |  |  |  |  |  |  |  |  |
|  | Per 10 min | 0.95 (0.90, 0.99) | 0.020 | 0.94 (0.89, 0.98) | 0.015 | 0.93 (0.88, 0.98) | 0.009 | 0.93 (0.88, 0.98) | 0.009 |
|  | Per 1 min | 0.99 (0.99, 1.00) | 0.020 | 0.99 (0.99, 1.00) | 0.015 | 0.99 (0.99, 1.00) | 0.009 | 0.99 (0.99, 1.00) | 0.009 |
| mRS 0-4 | |  |  |  |  |  |  |  |  |
|  | Per 10 min | 0.97 (0.94, 1.00) | 0.050 | 0.94 (0.90, 0.98) | 0.006 | 0.94 (0.89, 0.97) | 0.002 | 0.94 (0.90, 0.98) | 0.002 |
|  | Per 1 min | 1.00 (0.99, 1.00) | 0.050 | 0.99 (0.99, 1.00) | 0.006 | 0.99 (0.99, 1.00) | 0.002 | 0.99 (0.99, 1.00) | 0.002 |
| mRS 0-6 | |  |  |  |  |  |  |  |  |
|  | Per 10 min | 1.04 (1.01, 1.07) | 0.008 | 1.04 (1.01, 1.07) | <0.001 | 1.04 (1.01, 1.07) | <0.001 | 1.08 (1.04, 1.12) | <0.001 |
|  | Per 1 min | 1.004 (1.001, 1.007) | 0.004 | 1.006 (1.003, 1.009) | <0.001 | 1.007 (1.003, 1.010) | <0.001 | 1.008 (1.004, 1.012) | <0.001 |
| Mortality | |  |  |  | |  |  |  |  |
|  | Per 10 min | 1.03 (1.00, 1.07) | 0.040 | 1.06 (1.02, 1.10) | 0.004 | 1.07 (1.03, 1.12) | 0.001 | 1.07 (1.03, 1.12) | 0.001 |
|  | Per 1 min | 1.00 (1.00, 1.01) | 0.042 | 1.00 (1.00, 1.01) | 0.004 | 1.00 (1.00, 1.01) | 0.001 | 1.00 (1.00, 1.01) | 0.001 |
| sICH | |  |  |  |  |  |  |  |  |
|  | Per 10 min | 1.02 (0.98, 1.06) | 0.310 | 1.03 (0.98, 1.07) | 0.243 | 1.03 (0.98, 1.08) | 0.186 | 1.03 (0.99, 1.08) | 0.186 |
|  | Per 1 min | 1.00 (1.00, 1.01) | 0.310 | 1.00 (1.00, 1.01) | 0.243 | 1.00 (1.00, 1.01) | 0.186 | 1.00 (1.00, 1.01) | 0.186 |
| Notes: Analysis restricted to 472 patients with complete data (18 patients with missing values were excluded from the total cohort of 490). Data are odds ratios (ORs) with 95% confidence intervals (CIs) from complete-case analyses.  Procedure time was modelled as a continuous exposure, parameterised per 1-minute increase (PT) or per 10-minute increase (PT10), as indicated.  “Crude” denotes unadjusted models.  Model 1 was adjusted for age, baseline NIHSS score, ASPECTS, ASITN/SIR collateral grade, occlusion site, and TOAST subtype.  Model 2 was additionally adjusted for sex and admission glucose.  Model 3 was additionally adjusted for center as fixed effects (categorical).  For dichotomous functional outcomes (mRS 0–2, 0–3, and 0–4), the event was defined as a favourable outcome; therefore, ORs <1 indicate lower odds of achieving a favourable outcome with longer procedure time.  For the ordinal 90-day mRS (0–6) outcome, ORs >1 indicate a shift towards higher (worse) mRS categories with longer procedure time.  Abbreviations: mRS, modified Rankin Scale; sICH, symptomatic intracranial hemorrhage; OR, odds ratio; CI, confidence interval; NIHSS, National Institutes of Health Stroke Scale; ASPECTS, Alberta Stroke Program Early CT Score; ASITN/SIR, American Society of Interventional and Therapeutic Neuroradiology/Society of Interventional Radiology collateral grading system; TOAST, Trial of Org 10172 in Acute Stroke Treatment classification. | | | | | | | | | |

**eTable 6. Complete-case sensitivity analyses of categorical procedure time and outcomes in patients achieving successful reperfusion (mTICI ≥2b)**

| Outcome | | No (%) | crude OR | p value | Model 1 | | Model 2 | | Model 3 | |
| --- | --- | --- | --- | --- | --- | --- | --- | --- | --- | --- |
|  |  |  |  |  | Adjusted OR | p value | Adjusted OR | p value | Adjusted OR | p value |
| Primary outcome | |  |  |  |  |  |  |  |  |  |
| mRS 0-3 | |  |  |  |  |  |  |  |  |  |
|  | ≤ 60 min | 82/177 (46.3) | Reference | — | Reference | — | Reference | — | Reference | — |
|  | >60 to ≤ 120 min | 58/166 (34.9) | 0.58 (0.38, 0.91) | 0.016 | 0.47 (0.28, 0.79) | 0.005 | 0.47 (0.27, 0.80) | 0.006 | 0.46 (0.26, 0.80) | 0.006 |
|  | >120 min | 27/74 (36.5) | 0.62 (0.36, 1.09) | 0.098 | 0.37 (0.18, 0.74) | 0.005 | 0.36 (0.18, 0.74) | 0.005 | 0.35 (0.16, 0.74) | 0.006 |
|  | P for trend | P value =0.002 | | | | | | | | |
| Secondary outcome | |  |  |  |  |  |  |  |  |  |
| mRS 0-2 | |  |  |  |  |  |  |  |  |  |
|  | ≤ 60 min | 49/177  (27.7) | Reference | — | Reference | — | Reference | — | Reference | — |
|  | >60 to ≤ 120 min | 37/166 (22.3) | 0.71 (0.43, 1.16) | 0.169 | 0.72 (0.42, 1.23) | 0.228 | 0.76 (0.43, 1.31) | 0.32 | 0.76 (0.43, 1.31) | 0.32 |
|  | >120 min | 14/74 (18.9) | 0.58 (0.30, 1.13) | 0.112 | 0.51 (0.24, 1.07) | 0.075 | 0.52 (0.24, 1.11) | 0.091 | 0.52 (0.24, 1.11) | 0.091 |
|  | P for trend | P value =0.082 | | | | | | | | |
| mRS 0-4 | |  |  |  |  |  |  |  |  |  |
|  | ≤ 60 min | 101/177 (57.1) | Reference | — | Reference | — | Reference | — | Reference | — |
|  | >60 to ≤ 120 min | 84/166 (50.6) | 0.74 (0.48, 1.13) | 0.166 | 0.65 (0.39, 1.10) | 0.108 | 0.67 (0.39, 1.16) | 0.152 | 0.67 (0.39, 1.16) | 0.152 |
|  | >120 min | 37/74 (50.0) | 0.71 (0.41, 1.23) | 0.22 | 0.42 (0.21, 0.84) | 0.014 | 0.41 (0.20, 0.83) | 0.014 | 0.41 (0.20, 0.83) | 0.014 |
|  | P for trend | P value =0.013 | | | | | | | | |
| mRS 0 to 6 (median [IQR]) | |  |  |  |  |  |  |  |  |  |
|  | ≤ 60 min | 4 (2, 6) | Reference | — | Reference | — | Reference | — | Reference | — |
|  | >60 to ≤ 120 min | 4 (3, 6) | 1.46 (0.99, 2.14) | 0.056 | 1.51 (1.00, 2.27) | 0.048 | 1.47 (0.97, 2.21) | 0.068 | 1.47 (0.96, 2.25) | 0.074 |
|  | >120 min | 4 (3, 6) | 1.60 (0.98, 2.63) | 0.063 | 2.17 (1.26, 3.74) | 0.005 | 2.12 (1.22, 3.68) | 0.007 | 2.18 (1.23, 3.88) | 0.008 |
|  | P for trend | P value =0.006 | | | | | | | | |
| Mortality | |  |  |  |  | |  |  |  |  |
|  | ≤ 60 min | 64/177 (36.2) | Reference | — | Reference | — | Reference | — | Reference | — |
|  | >60 to ≤ 120 min | 66/166 (39.8) | 1.26 (0.81, 1.96) | 0.31 | 1.41 (0.84, 2.37) | 0.199 | 1.33 (0.77, 2.28) | 0.302 | 1.33 (0.77, 2.31) | 0.303 |
|  | >120 min | 33/74 (44.6) | 1.53 (0.88, 2.67) | 0.135 | 2.53 (1.28, 5.01) | 0.008 | 2.58 (1.28, 5.22) | 0.008 | 2.65 (1.28, 5.52) | 0.009 |
|  | P for trend | P value =0.013 | | | | | | | | |
| sICH | |  |  |  |  |  |  |  |  |  |
|  | ≤ 60 min | 20/177 (11.3) | Reference | — | Reference | — | Reference | — | Reference | — |
|  | >60 to ≤ 120 min | 23/166 (13.9) | 1.25 (0.65, 2.40) | 0.507 | 1.31 (0.66, 2.59) | 0.441 | 1.22 (0.61, 2.45) | 0.566 | 1.22 (0.61, 2.45) | 0.566 |
|  | >120 min | 12/74 (16.2) | 1.55 (0.71, 3.38) | 0.272 | 1.60 (0.69, 3.70) | 0.271 | 1.58 (0.68, 3.70) | 0.289 | 1.58 (0.68, 3.70) | 0.289 |
|  | P for trend | P value =0.290 | | | | | | | | |
| Note: Data are odds ratios (ORs) with 95% confidence intervals (CIs) derived from complete-case analyses restricted to patients achieving successful reperfusion (mTICI ≥2b). N Outcome counts [No. (%)] are calculated among patients with successful reperfusion and observed PT (n=417). Regression models were fitted in complete cases for model covariates (missing glucose were excluded, analytic n=408);  For binary functional outcomes (mRS 0–2, 0–3, and 0–4) and safety outcomes (mortality and sICH), the “No. (%)” column reports the number and percentage of patients with the outcome in each procedure-time group;  for the ordinal 90-day mRS (0–6) outcome, this column reports the median (IQR) and ORs are from cumulative logit proportional-odds (shift) models.  Procedure time was categorized as ≤60, >60 to ≤120 min, and >120 minutes, with ≤60 minutes as the reference (OR=1.00).  “Crude” denotes unadjusted models.  Model 1 was adjusted for age, baseline NIHSS score, ASPECTS, ASITN/SIR collateral grade, occlusion site, and TOAST subtype.  Model 2 was additionally adjusted for sex and admission glucose.  Model 3 additionally included a random intercept for center (see eMethods S6.3); mixed-effects logistic regression was used for binary outcomes and mixed-effects proportional-odds models for the ordinal mRS shift outcome. P for trend was obtained from the Wald test by modeling procedure-time category as an ordinal variable (1, 2, 3) with the same fixed- and random-effects structure as Model 3.  For dichotomous functional outcomes, the event was defined as a favorable outcome; thus, ORs <1 indicate lower odds of achieving a favorable outcome with longer procedure time. For the ordinal mRS (0–6) shift outcome, ORs >1 indicate a shift toward higher (worse) mRS categories. Abbreviations: mRS, modified Rankin Scale; sICH, symptomatic intracranial hemorrhage; OR, odds ratio; CI, confidence interval; PT, procedure time; ASPECTS, Alberta Stroke Program Early CT Score; NIHSS, National Institutes of Health Stroke Scale; TOAST, Trial of Org 10172 in Acute Stroke Treatment classification; ASITN/SIR, American Society of Interventional and Therapeutic Neuroradiology/Society of Interventional Radiology collateral grading system; EVT, endovascular thrombectomy. | | | | | | | | | | |

**eTable 7. Complete-case sensitivity analyses of continuous procedure time and outcomes in patients achieving successful reperfusion (mTICI ≥2b)**

| Outcome | | crude OR | p value | Model 1 | | Model 2 | | Model 3 | |
| --- | --- | --- | --- | --- | --- | --- | --- | --- | --- |
|  |  |  |  | Adjusted OR | p value | Adjusted OR | p value | Adjusted OR | p value |
| Primary outcome | |  |  |  |  |  |  |  |  |
| mRS 0-3 | |  |  |  |  |  |  |  |  |
|  | Per 10 min | 0.96 (0.92, 1.00) | 0.029 | 0.92 (0.88, 0.97) | 0.002 | 0.92 (0.87, 0.97) | 0.002 | 0.92 (0.87, 0.97) | 0.002 |
|  | Per 1 min | 1.00 (0.99, 1.00) | 0.029 | 0.99 (0.99, 1.00) | 0.002 | 0.99 (0.99, 1.00) | 0.002 | 0.99 (0.99, 1.00) | 0.002 |
| mRS 0-2 | |  |  |  |  |  |  |  |  |
|  | Per 10 min | 0.96 (0.92, 1.01) | 0.086 | 0.95 (0.90, 1.00) | 0.075 | 0.95 (0.90, 1.01) | 0.079 | 0.95 (0.90, 1.01) | 0.079 |
|  | Per 1 min | 1.00 (0.99, 1.00) | 0.086 | 1.00 (0.99, 1.00) | 0.075 | 1.00 (0.99, 1.00) | 0.079 | 1.00 (0.99, 1.00) | 0.079 |
| mRS 0-4 | |  |  |  |  |  |  |  |  |
|  | Per 10 min | 0.97 (0.94, 1.01) | 0.094 | 0.94 (0.90, 0.99) | 0.012 | 0.94 (0.89, 0.98) | 0.007 | 0.94 (0.89, 0.98) | 0.007 |
|  | Per 1 min | 1.00 (0.99, 1.00) | 0.094 | 0.99 (0.99, 1.00) | 0.012 | 0.99 (0.99, 1.00) | 0.007 | 0.99 (0.99, 1.00) | 0.007 |
| mRS 0 to 6 | |  |  |  |  |  |  |  |  |
|  | Per 10 min | 1.04 (1.01, 1.07) | 0.022 | 1.06 (1.02, 1.10) | 0.003 | 1.06 (1.02, 1.10) | 0.002 | 1.06 (1.02, 1.10) | 0.002 |
|  | Per 1 min | 1.00 (1.00, 1.01) | 0.022 | 1.01 (1.00, 1.01) | 0.003 | 1.01 (1.00, 1.01) | 0.002 | 1.01 (1.00, 1.01) | 0.002 |
| Mortality | |  |  |  |  |  |  |  |  |
|  | Per 10 min | 1.03 (1.00, 1.07) | 0.057 | 1.07 (1.02, 1.11) | 0.006 | 1.07 (1.02, 1.12) | 0.003 | 1.07 (1.02, 1.12) | 0.003 |
|  | Per 1 min | 1.00 (1.00, 1.01) | 0.057 | 1.01 (1.00, 1.01) | 0.006 | 1.01 (1.00, 1.01) | 0.003 | 1.01 (1.00, 1.01) | 0.003 |
| sICH | |  |  |  |  |  |  |  |  |
|  | Per 10 min | 1.02 (0.98, 1.07) | 0.381 | 1.02 (0.98, 1.07) | 0.353 | 1.02 (0.97, 1.08) | 0.357 | 1.02 (0.97, 1.08) | 0.357 |
|  | Per 1 min | 1.00 (1.00, 1.01) | 0.381 | 1.00 (1.00, 1.01) | 0.353 | 1.00 (1.00, 1.01) | 0.357 | 1.00 (1.00, 1.01) | 0.357 |
| Note: Data are odds ratios (ORs) with 95% confidence intervals (CIs) derived from complete-case analyses restricted to patients achieving successful reperfusion (mTICI ≥2b). Procedure time (PT) was modeled either per 10-minute increase (PT/10) or per 1-minute increase (PT, in minutes).  “Crude” denotes unadjusted models including PT only.  Model 1 was adjusted for age, baseline NIHSS score, ASPECTS, ASITN/SIR collateral grade, occlusion site, and TOAST subtype.  Model 2 was additionally adjusted for sex and admission glucose.  Model 3 additionally included a random intercept for center (see eMethods S6.3); mixed-effects logistic regression was used for binary outcomes and mixed-effects proportional-odds models for the ordinal mRS 0–6 shift outcome. For dichotomous functional outcomes (mRS 0–2, 0–3, and 0–4), the event was defined as a favorable outcome; thus, ORs <1 indicate lower odds of achieving a favorable outcome with longer procedure time.  For the ordinal mRS 0–6 shift outcome, ORs >1 indicate a shift toward higher (worse) mRS categories with longer procedure time.  For mortality and sICH, ORs >1 indicate increased odds of the respective adverse outcome with longer procedure time. Abbreviations: mRS, modified Rankin Scale; sICH, symptomatic intracranial hemorrhage; OR, odds ratio; CI, confidence interval; PT, procedure time; ASPECTS, Alberta Stroke Program Early CT Score; NIHSS, National Institutes of Health Stroke Scale; TOAST, Trial of Org 10172 in Acute Stroke Treatment classification; ASITN/SIR, American Society of Interventional and Therapeutic Neuroradiology/Society of Interventional Radiology collateral grading system. | | | | | | | | | |

**eTable 8. Reference-patient–based adjusted probabilities and absolute risk differences at key procedure times for 90-day mRS 0–3 and mortality**

| Procedure time | mRS 0-3 at 90 days | | Mortality at 90 days | |
| --- | --- | --- | --- | --- |
|  | Adjusted Probability (95% CI) | ARD vs 60 min, percentage points (95% CI) | Adjusted Probability (95% CI) | ARD vs 60 min, percentage points (95% CI) |
| 60 minutes | 42.2% (25.9 – 58.6) | Reference | 30.9% (16.9 – 44.9) | Reference |
| 90 minutes | 33.3% (18.6 – 48.0) | -9.0 (-17.3, -0.6) | 33.8% (19.5 – 48.0) | 2.9 (-4.0, 9.7) |
| 120 minutes | 25.1% (10.9 – 39.3) | -17.2 (-30.9, -3.4) | 36.0% (19.8 – 52.3) | 5.1 (-7.9, 18.1) |
| 150 minutes | 19.5% (6.7 – 32.3) | -22.7 (-37.2, -8.3) | 39.8% (21.9 – 57.7) | 8.9 (-6.7, 24.5) |
| Note: Values are reference-patient–based adjusted outcome probabilities and absolute risk differences (ARDs) at selected procedure times, estimated from mixed-effects logistic regression models with a random intercept for center.  Models were fitted within 20 multiply imputed datasets (n=490) and pooled using Rubin’s rules.  Procedure time was modeled per 10 minutes (PT10) using restricted cubic splines with 4 knots at the 5th, 35th, 65th, and 95th percentiles of the observed PT10 distribution.  Models were adjusted for age, baseline NIHSS score, ASPECTS, ASITN/SIR collateral grade, occlusion site, TOAST subtype, sex, and admission glucose.  The reference patient was defined using the median values of continuous covariates and the most frequent categories of categorical covariates;  predictions were based on fixed effects with the center-specific random intercept set to 0. ARDs are expressed as percentage-point differences in outcome probability compared with PT=60 minutes.  For mRS 0–3, negative ARDs indicate a lower probability of favorable functional outcome with longer procedure time; for mortality, positive ARDs indicate a higher probability of death.  Abbreviations: ARD, absolute risk difference; ASPECTS, Alberta Stroke Program Early CT Score; CI, confidence interval; mRS, modified Rankin Scale; NIHSS, National Institutes of Health Stroke Scale; TOAST, Trial of Org 10172 in Acute Stroke Treatment; ASITN/SIR, American Society of Interventional and Therapeutic Neuroradiology/Society of Interventional Radiology. | | | | |

**eTable 9. Baseline and procedural characteristics among patients with procedure time ≤60 minutes, stratified by <30 versus 30–60 minutes.**

|  | | <30 min  (n = 30) | 30-60 min  (n = 155) | SMD |
| --- | --- | --- | --- | --- |
| Age, y, median (IQR) | | 67 (60-76) | 69 (59-78) | 0.056 |
| Sex, male, n () | | 17 (56.7) | 92 (59.4) | 0.054 |
| Glucose, mmol/L, median (IQR) | | 7.6 (5.8-8.9) | 6.9 (5.8-8.5) | 0.078 |
| BP, mmHg, median (IQR) | | |  |  |
|  | Systolic | 134 (121-150) | 145 (130-160) | 0.395 |
|  | Diastolic | 78 (69-87) | 86 (77-96) | 0.495 |
| Medical history, n () | |  |  |  |
|  | Hypertension | 15 (50.0) | 99 (63.9) | 0.280 |
|  | Hyperlipidemia | 6 (20.0) | 40 (25.8) | 0.138 |
|  | Diabetes | 5 (16.7) | 24 (15.5) | 0.032 |
|  | Smoking | 7 (23.3) | 56 (36.1) | 0.280 |
|  | Atrial fibrillation | 11 (36.7) | 83 (53.5) | 0.339 |
| ASPECTS, median (IQR) | | 4 (3-5) | 4 (2-5) | 0.006 |
| NIHSS score, median (IQR) | | 18 (16-20) | 17 (14-20) | 0.133 |
| ASITN/SIR, n () | |  |  | 0.514 |
|  | 0–1 | 11 (36.7) | 83 (53.5) |  |
|  | 2 | 9 (30.0) | 52 (33.5) |  |
|  | 3-4 | 10 (33.3) | 20 (12.9) |  |
| TOAST, n () | |  |  | 0.445 |
|  | LAA | 19 (63.3) | 106 (68.4) |  |
|  | CE | 4 (13.3) | 34 (21.9) |  |
|  | Others/unknown | 7 (23.3) | 15 (9.7) |  |
| Occlusion site, n () | |  |  | 0.400 |
|  | ICA | 8 (26.7) | 60 (38.7) |  |
|  | M1 segment | 20 (66.7) | 81 (52.3) |  |
|  | M2 segment | 2 (6.7) | 14 (9.0) |  |
| First choice of EVT, n () | | |  | 0.326 |
|  | Stent thrombectomy | 13 (43.3) | 49 (31.6) |  |
|  | Aspiration | 15 (50.0) | 94 (60.6) |  |
|  | Others | 2 (6.7) | 12 (7.7) |  |
| IVT, n () | | 26 (86.7) | 118 (76.1) | 0.271 |
| OTI, min, median (IQR) | | 358 (232-604) | 271 (164-392) | 0.379 |
| OTP, min, median (IQR) | | 443 (326-689) | 348 (230-468) | 0.584 |
| Anesthesia, general, n () | | 20 (66.7) | 125 (80.6) | 0.317 |
| Values are presented as median (interquartile range) for continuous variables and n/N () for categorical variables. Patients were restricted to those with observed procedure time ≤60 minutes and stratified into PT <30 minutes and PT 30–60 minutes. Standardized mean differences (SMDs) are shown to describe between-group imbalance. For binary variables displayed with two levels and for categorical variables with more than two levels, SMDs are presented at the variable level rather than separately for each category. This exploratory analysis was performed to assess potential heterogeneity within the original ≤60-minute reference category.  Abbreviations: ASITN/SIR, American Society of Interventional and Therapeutic Neuroradiology/Society of Interventional Radiology; ASPECTS, Alberta Stroke Program Early CT Score; CE, cardioembolism; EVT, endovascular thrombectomy; ICA, internal carotid artery; IQR, interquartile range; IVT, intravenous thrombolysis; LAA, large-artery atherosclerosis; M1, M1 segment of the middle cerebral artery; M2, M2 segment of the middle cerebral artery; NIHSS, National Institutes of Health Stroke Scale; PT, procedure time; SMD, standardized mean difference; TOAST, Trial of Org 10172 in Acute Stroke Treatment. | | | | |

**eTable 10. Exploratory outcome analysis among patients with procedure time ≤60 minutes, stratified by <30 versus 30–60 minutes.**

| Outcome | | <30 min (n=30) | 30-60 min (n=155) |  | | Model1 | | Model2 | |
| --- | --- | --- | --- | --- | --- | --- | --- | --- | --- |
|  |  |  |  | Crude OR (95%CI) | P value | Adjusted OR (95% CI) | P value | Adjusted OR (95%CI) | P value |
| 90-day ordinal mRS shift | | 4 (3-6) | 4 (2-6) | 1.15 (0.58-2.27) | 0.687 | 0.84 (0.38-1.88) | 0.676 | 1.12 (0.49-2.54) | 0.784 |
|  | mRS 0-2 | 7 (23.3) | 42 (27.1) | 1.22 (0.49-3.06) | 0.669 | 1.59 (0.56-4.55) | 0.384 | 1.37 (0.47-4.03) | 0.562 |
|  | mRS 0-3 | 11 (36.7) | 72 (46.5) | 1.50 (0.67-3.36) | 0.326 | 3.07 (1.03-9.15) | 0.044 | 2.75 (0.85-8.85) | 0.091 |
|  | mRS 0-4 | 19 (63.3) | 85 (54.8) | 0.70 (0.31-1.58) | 0.392 | 0.83 (0.29-2.39) | 0.735 | 0.72 (0.23-2.20) | 0.560 |
| mortality | | 8 (26.7) | 60 (38.7) | 1.74 (0.73-4.15) | 0.214 | 1.58 (0.50-4.98) | 0.434 | 2.28 (0.63-8.25) | 0.209 |
| sICH | | 6 (20.0) | 15 (9.7) | 0.43 (0.15-1.21) | 0.111 | 0.30 (0.08-1.13) | 0.075 | 0.28 (0.07-1.05) | 0.059 |
| Values are presented as median (interquartile range) for the ordinal mRS score and n (%) for binary outcomes. Patients were restricted to those with observed procedure time ≤60 minutes and were stratified into PT <30 minutes and PT 30–60 minutes. The reference group was PT <30 minutes; therefore, odds ratios compare PT 30–60 minutes with PT <30 minutes. For favourable functional outcomes (mRS 0–2, mRS 0–3, and mRS 0–4), OR <1 indicates lower odds of achieving the outcome in the 30–60-minute group. For mortality and sICH, OR >1 indicates higher risk in the 30–60-minute group. For ordinal mRS shift, common OR >1 indicates a shift toward worse mRS scores in the 30–60-minute group. Crude models included only the PT subgroup variable. Model 1 adjusted for age, baseline NIHSS score, ASPECTS, ASITN/SIR collateral grade, occlusion site, and TOAST subtype. Model 2 additionally adjusted for sex and admission glucose. Analyses were exploratory and based on observed complete data within the original ≤60-minute reference category.  Abbreviations: ASITN/SIR, American Society of Interventional and Therapeutic Neuroradiology/Society of Interventional Radiology; ASPECTS, Alberta Stroke Program Early CT Score; CI, confidence interval; mRS, modified Rankin Scale; NIHSS, National Institutes of Health Stroke Scale; OR, odds ratio; PT, procedure time; sICH, symptomatic intracranial hemorrhage; TOAST, Trial of Org 10172 in Acute Stroke Treatment. | | | | | | | | | |

**eTable 11. Exploratory PT-by-collateral interaction analysis in the overall EVT cohort.**

| **Outcome** | **Collateral stratum** | **Stratum N** | **OR per 10-min PT increase (95% CI)** | **P value** | **P for interaction** |
| --- | --- | --- | --- | --- | --- |
| 90-day ordinal mRS shift | Poor collaterals (ASITN/SIR 0–1) | 239 | 1.03 (0.98–1.09) | 0.244 | 0.186 |
|  | Better collaterals (ASITN/SIR 2–4) | 251 | 1.08 (1.04–1.12) | <0.001 |  |
| 90-day mRS 0–2 | Poor collaterals (ASITN/SIR 0–1) | 239 | 0.88 (0.76–1.01) | 0.061 | 0.268 |
|  | Better collaterals (ASITN/SIR 2–4) | 251 | 0.95 (0.90–1.00) | 0.071 |  |
| 90-day mRS 0–3 | Poor collaterals (ASITN/SIR 0–1) | 239 | 0.95 (0.87–1.03) | 0.192 | 0.291 |
|  | Better collaterals (ASITN/SIR 2–4) | 251 | 0.90 (0.85–0.95) | <0.001 |  |
| 90-day mRS 0–4 | Poor collaterals (ASITN/SIR 0–1) | 239 | 0.97 (0.90–1.04) | 0.433 | 0.249 |
|  | Better collaterals (ASITN/SIR 2–4) | 251 | 0.92 (0.88–0.97) | 0.003 |  |
| 90-day mortality | Poor collaterals (ASITN/SIR 0–1) | 239 | 1.01 (0.95–1.08) | 0.748 | 0.047 |
|  | Better collaterals (ASITN/SIR 2–4) | 251 | 1.10 (1.04–1.16) | <0.001 |  |
| sICH | Poor collaterals (ASITN/SIR 0–1) | 239 | 1.03 (0.97–1.09) | 0.295 | 0.930 |
|  | Better collaterals (ASITN/SIR 2–4) | 251 | 1.03 (0.96–1.10) | 0.450 |  |
| **Note:** Collateral status was dichotomized as poor collaterals (ASITN/SIR 0–1) versus better collaterals (ASITN/SIR 2–4, including intermediate and good collateral status) to preserve model stability and to evaluate whether collateral status modified the association between PT and outcomes. PT was modeled continuously per 10-minute increase. Models included PT10, collateral group, the PT10-by-collateral interaction term, age, baseline NIHSS score, ASPECTS, occlusion site, TOAST subtype, sex, and admission glucose. Models were fitted separately in each of the 20 imputed datasets and pooled using Rubin’s rules. ORs represent the association between each 10-minute increase in PT and the outcome within each collateral stratum. P for interaction tests whether the PT–outcome association differed between poor and better collateral strata. For favourable functional outcomes, OR <1 indicates lower odds of achieving the outcome with longer PT. For mortality and sICH, OR >1 indicates higher risk with longer PT. For ordinal mRS shift, common OR >1 indicates a shift toward worse mRS scores with longer PT.  **Abbreviations:** ASITN/SIR, American Society of Interventional and Therapeutic Neuroradiology/Society of Interventional Radiology; ASPECTS, Alberta Stroke Program Early CT Score; CI, confidence interval; mRS, modified Rankin Scale; NIHSS, National Institutes of Health Stroke Scale; OR, odds ratio; PT, procedure time; sICH, symptomatic intracranial hemorrhage; TOAST, Trial of Org 10172 in Acute Stroke Treatment. | | | | | |

**Supplementary Figures**

**eFigure S1. Directed Acyclic Graph (DAG) of the Causal Structure Between Procedure Time (PT) and 90-Day Outcomes.**


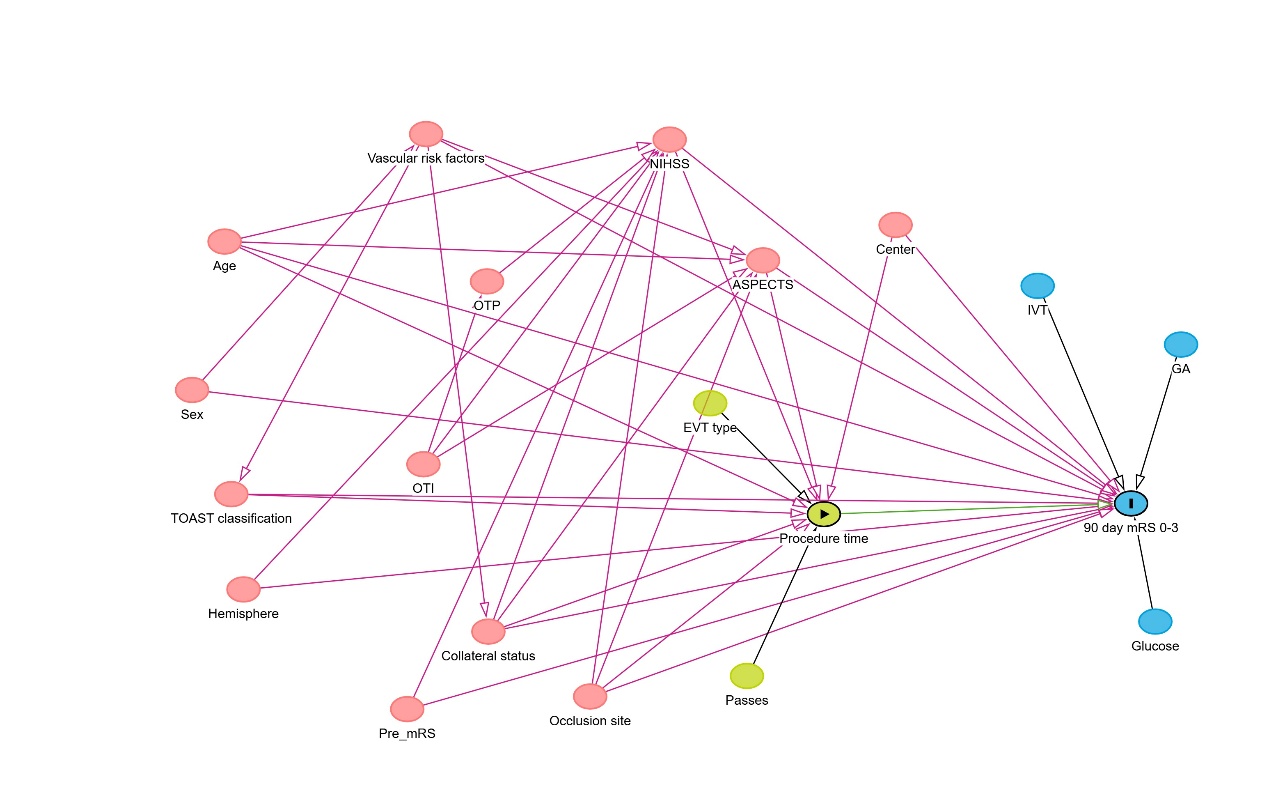

This figure illustrates the assumed causal structure between PT and the 90-day favorable functional outcome (mRS 0–3), along with the rationale for covariate selection. Pink nodes represent potential confounders (baseline severity, infarct extent, collateral status, occlusion site, TOAST classification, age, and center). Yellow nodes represent potential mediators in the "PT → outcome" pathway (e.g., thrombectomy strategy and number of passes). Blue nodes represent variables related to treatment or prognosis that were not included in the minimally sufficient adjustment set (e.g., IVT, general anesthesia, and admission glucose). Based on the backdoor criterion, the minimally sufficient adjustment set for estimating the association between PT and 90-day outcomes includes: age, baseline NIHSS, ASPECTS, ASITN/SIR collateral grade, occlusion site, TOAST classification, and center.

**eFigure S2. Missing Data for Key Continuous Variables**


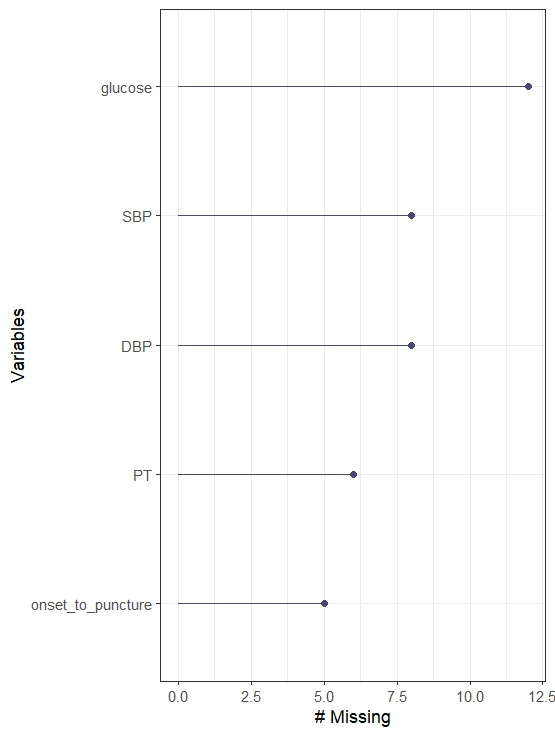


**Figure Caption:** This figure shows the number of missing observations for the continuous variables included in the multiple imputation model (admission glucose, systolic blood pressure, diastolic blood pressure, procedure time [PT], and onset-to-puncture time).

**eFigure S3. Distribution Comparison of Observed vs. Imputed Values for Continuous Variables**


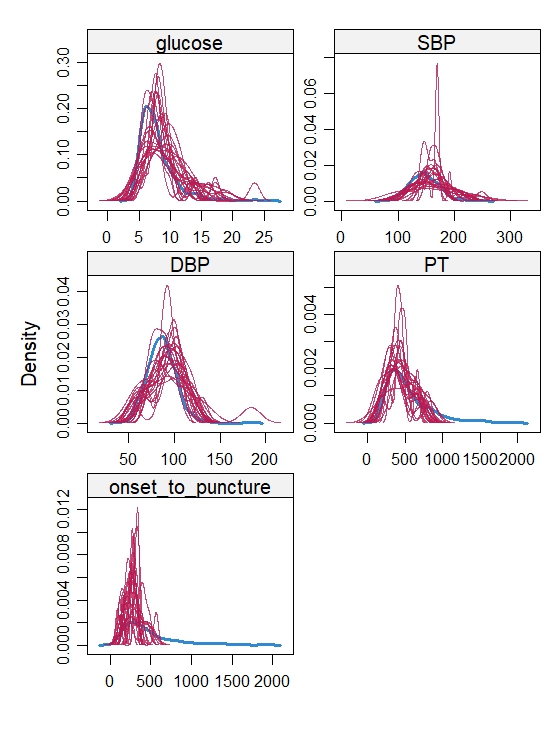


**Figure Caption:** The kernel density curves compare the distribution of observed values (blue curve) and imputed values (pink curve) for admission glucose, systolic blood pressure, diastolic blood pressure, procedure time (PT), and onset-to-puncture time across 20 imputed datasets. The distribution of imputed values is generally consistent with the observed data and within the reasonable range of values.

**eFigure S4. Internal Diagnostic Plot for 20 Imputed Datasets**


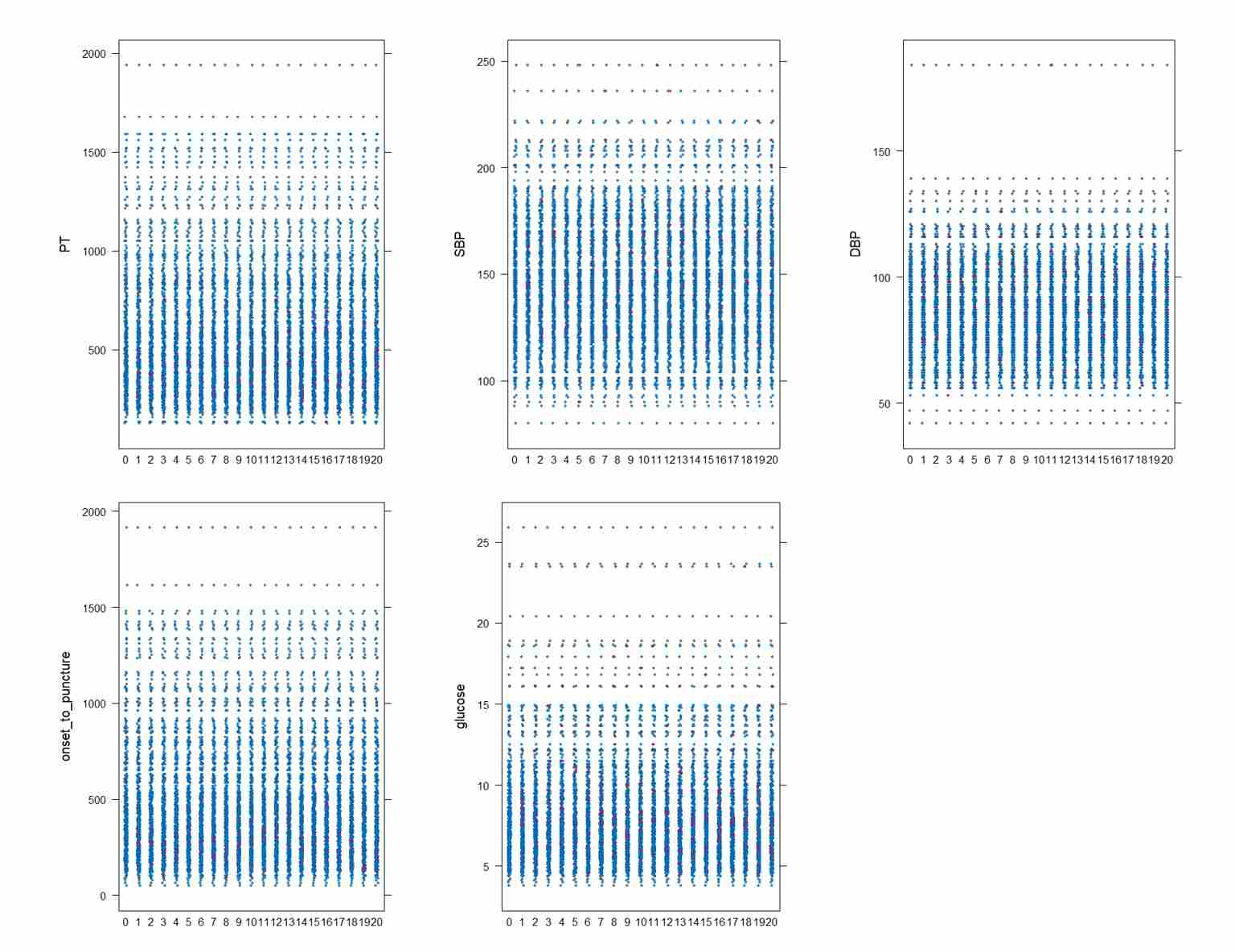


**Figure Caption:** The plot displays the observed values (blue dots) and imputed values (pink dots) for admission glucose, systolic blood pressure, diastolic blood pressure, procedure time (PT), and onset-to-puncture time across 20 imputed datasets (x-axis). The distribution of imputed values closely matches the observed values along the y-axis, with the points interspersed, indicating that the imputed results are reasonable and do not introduce any significant outliers.

**eFigure S5. Adjusted predicted probabilities of 90-day outcomes across procedure time.**


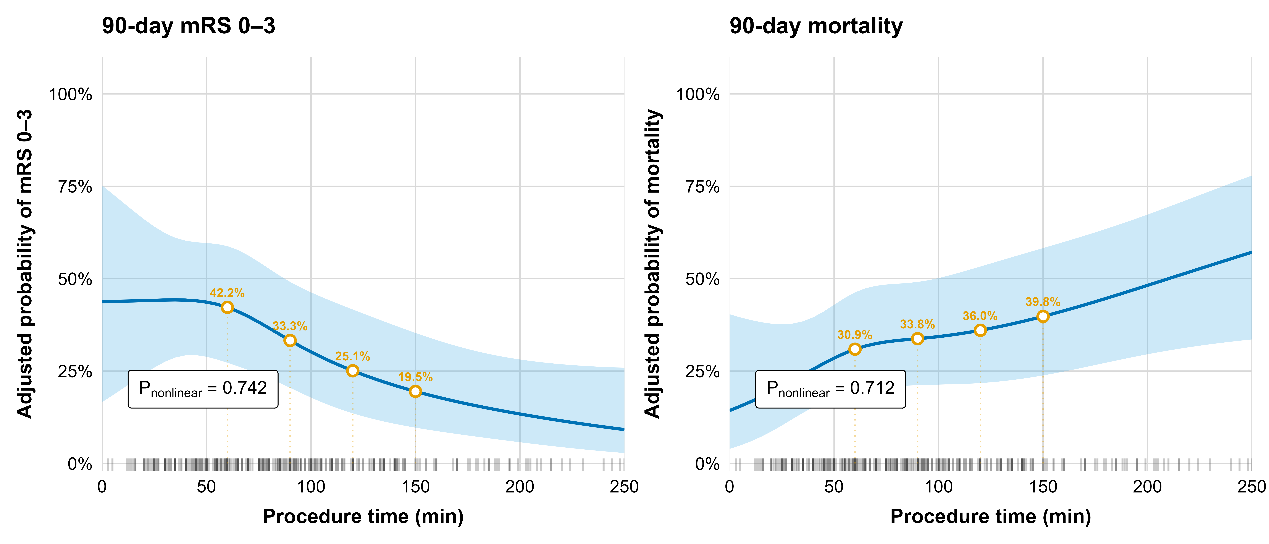


Adjusted predicted probabilities (solid line) with 95% confidence intervals (shaded band) are shown for (A) 90-day mRS 0–3 and (B) 90-day all-cause mortality across procedure time (PT, minutes). Predictions were obtained from fully adjusted mixed-effects logistic regression models with PT modeled per 10 minutes using restricted cubic splines (knots at the 5th, 35th, 65th, and 95th percentiles), with center included as a random intercept. Estimates were fitted within 20 multiply imputed datasets and pooled using Rubin’s rules. Predicted probabilities were generated for a reference patient (continuous covariates fixed at the median and categorical covariates fixed at the modal category; center random effect set to 0) while varying PT over a prespecified grid. P values for nonlinearity (insets) were obtained from Wald tests of the nonlinear spline components. Orange markers denote predicted probabilities at PT = 60, 90, 120, and 150 minutes; tick marks along the x-axis indicate the distribution of observed PT.

**eFigure S6. Covariate balance before and after inverse probability of treatment weighting across procedure time categories.**


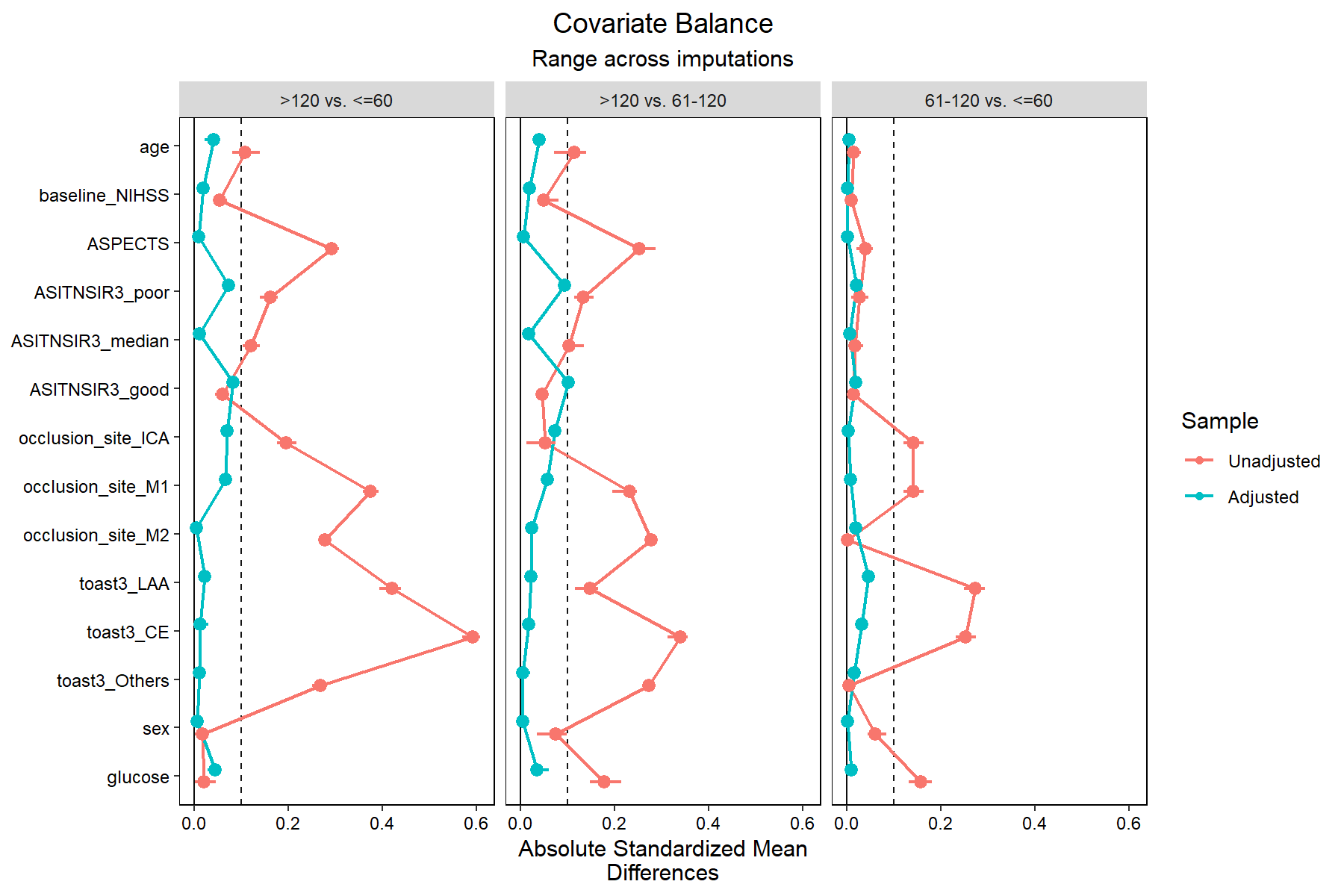


Love plots display absolute standardized mean differences (SMDs) for covariates used in the multinomial propensity score model (Model 2 covariate set) when comparing procedure time (PT) categories (≤60, 61–120, and >120 minutes). Each panel shows a pairwise comparison (>120 vs ≤60, >120 vs 61–120, and 61–120 vs ≤60). Red points/lines indicate SMDs in the unweighted sample, and blue points/lines indicate SMDs after applying inverse probability of treatment weighting (IPTW) for the average treatment effect. For each covariate, the plotted range summarizes SMDs across the 20 imputed datasets. The vertical dashed line at |SMD| = 0.10 denotes the prespecified balance criterion.
